# Early-life microbiota transplantation affects behavioural responses, serotonin and immune characteristics in chicken lines divergently selected on feather pecking

**DOI:** 10.1038/s41598-020-59125-w

**Published:** 2020-02-17

**Authors:** Jerine A. J. van der Eijk, T. Bas Rodenburg, Hugo de Vries, Joergen B. Kjaer, Hauke Smidt, Marc Naguib, Bas Kemp, Aart Lammers

**Affiliations:** 1 Behavioural Ecology Group, Department of Animal Sciences, Wageningen University and Research, Wageningen, the Netherlands; 2 Adaptation Physiology Group, Department of Animal Sciences, Wageningen University and Research, Wageningen, the Netherlands; 3 Department of Animals in Science and Society, Faculty of Veterinary Medicine, Utrecht University, Utrecht, the Netherlands; 4 Laboratory of Microbiology, Wageningen University and Research, Wageningen, the Netherlands; 5 Friedrich-Loeffler-Institut, Institute of Animal Welfare and Animal Husbandry, Celle, Germany

**Keywords:** Animal behaviour, Animal physiology, Immunology, Microbiology, Neuroscience

## Abstract

Gut microbiota influences host behaviour and physiology, such as anxiety, stress, serotonergic and immune systems. These behavioural and physiological characteristics are related to feather pecking (FP), a damaging behaviour in chickens that reduces animal welfare and productivity. Moreover, high FP (HFP) and low FP (LFP) lines differed in microbiota composition. However, it is unknown whether microbiota can influence the development of FP. For the first time, we identified the effects of microbiota transplantation on FP, and behavioural and physiological characteristics related to FP. HFP and LFP chicks received sterile saline (control), HFP or LFP microbiota transplantation during the first two weeks post-hatch. Microbiota transplantation influenced behavioural responses of the HFP line during treatment and of the LFP line after treatment. In both lines, homologous microbiota transplantation (i.e., receiving microbiota from their line) resulted in more active behavioural responses. Furthermore, microbiota transplantation influenced immune characteristics (natural antibodies) in both lines and peripheral serotonin in the LFP line. However, limited effects on microbiota composition, stress response (corticosterone) and FP were noted. Thus, early-life microbiota transplantation had immediate and long-term effects on behavioural responses and long-term effects on immune characteristics and peripheral serotonin; however, the effects were dependent on host genotype. Since early-life microbiota transplantation influenced behavioural and physiological characteristics that are related to FP, it could thus influence the development of FP later in life.

## Introduction

Early-life is crucial for an animal’s behavioural and physiological development and early-life factors can have a profound impact on this development^1^. An important moment early in life is the rapid microbial colonization of the gut, leading to the establishment of the gut microbiota. The gut microbiota influences host behaviour and physiology^2–5^. Furthermore, altering microbiota composition, via for example anti- or probiotic treatment, affects anxiety, stress and activity^6–8^, as well as the serotonergic and immune systems in rodents^9–12^. Moreover, germ-free mice colonized with microbiota from another mouse strain exhibit behavioural profiles of the donor strain^13^. The gut microbiota seems to have similar effects in poultry, where altering microbiota composition affects fearfulness, memory, and serotonergic and immune systems^14–16^. Moreover, microbiota transplantation to germ-free quails has resulted in recipients adopting the fearful behaviour of donors early in life; however, this effect reversed later in life^17^. These findings suggest that the gut microbiota influences behavioural and physiological characteristics in poultry and could therefore influence a bird’s ability to cope with environmental and social challenges, such as those encountered in animal production systems.

Excessive damaging behaviours are indicative of an animal’s inability to cope with a restrictive environment and are frequently seen in production animals. Feather pecking (FP) in chickens is one such damaging behaviour, which involves hens pecking and pulling at feathers of conspecifics, thereby reducing animal welfare and productivity^18^. Feather pecking is multifactorial and has been linked to numerous behavioural characteristics, such as fearfulness, stress and activity, as well as physiological characteristics, such as serotonergic, dopaminergic and immune systems^19–21^. Since behavioural and physiological systems that are related to FP are also affected by the gut microbiota, microbiota might play a role in the development of FP. Indeed, lines selected for high FP (HFP) and low FP (LFP) differ in behavioural responses, stress response, activity, central serotonergic and dopaminergic activity, peripheral serotonin, innate and adaptive immune characteristics^22–28^. Moreover, the HFP and LFP lines differ in intestinal microbial metabolites and microbiota composition determined from caecal droppings and intestinal luminal content^29–31^. These findings point to a relationship between the gut microbiota and FP, however, it is unknown whether the gut microbiota influences the development of FP.

Therefore, this study aims to identify the effects of early-life microbiota transplantation on FP and behavioural and physiological characteristics related to FP in lines divergently selected for FP (HFP and LFP lines). We further identify the effects of microbiota transplantation on microbiota composition. We hypothesize that microbiota transplantation results in recipients adopting a similar behavioural profile as that seen in the donor line. For example, LFP birds receiving HFP microbiota show more FP and more active behavioural responses compared to LFP birds receiving LFP microbiota or control treatment.

## Results

### High and low feather pecking transplantation pools had distinct microbiota composition

**Figure 1:**
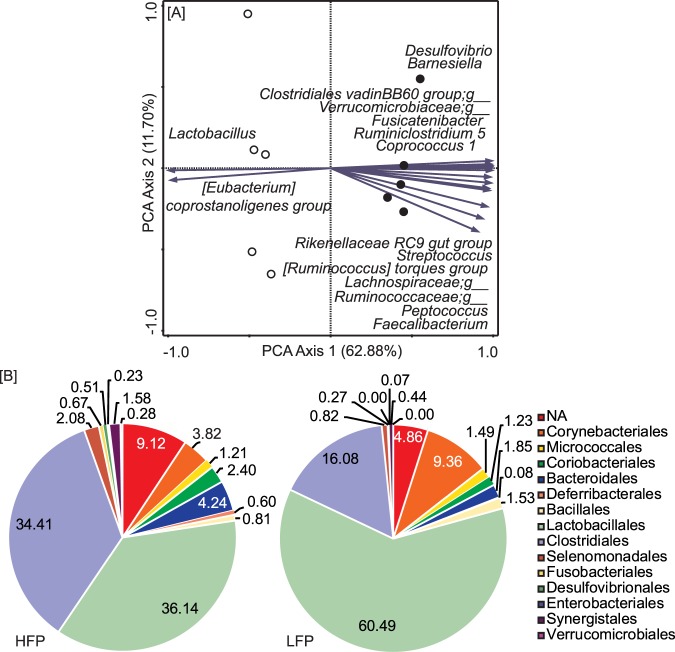
(**A**) Biplot for principal component analysis (PCA) of transplantation pools’ microbiota composition. Samples are grouped by line: HFP (closed circles) and LFP (open circles) with 5 replicates per pool. Microbial groups for which the variation in relative abundance in the data is explained for at least 95% by the axes are represented as vectors. Groups that could not be assigned to a specific genus are classified by the family name appended with “;g_”. (**B**) Pie charts with average relative abundances of orders present in the HFP (left) and LFP (right) transplantation pools.

Gut microbiota was collected from adult chickens of the HFP and LFP lines that were shown to differ in microbiota composition^31^. Transplantation pools were made per line and could be distinguished from each other in terms of microbiota composition using a principal component analysis (PCA) (Fig. 1A). The orders of Clostridiales and Lactobacillales had the highest relative abundance in both pools. The HFP pool had a higher relative abundance of Clostridiales and the LFP pool had a higher relative abundance of Lactobacillales (Fig. 1B). The number of viable microorganisms in the pools was analysed using plate cultures, and both pools contained on average 4.75 × 10^6^ viable aerobic colony forming units/mL and 5.1 × 10^6^ viable anaerobic colony forming units/mL.

### Early-life microbiota transplantation had limited effects on recipients’ microbiota composition

**Figure 2:**
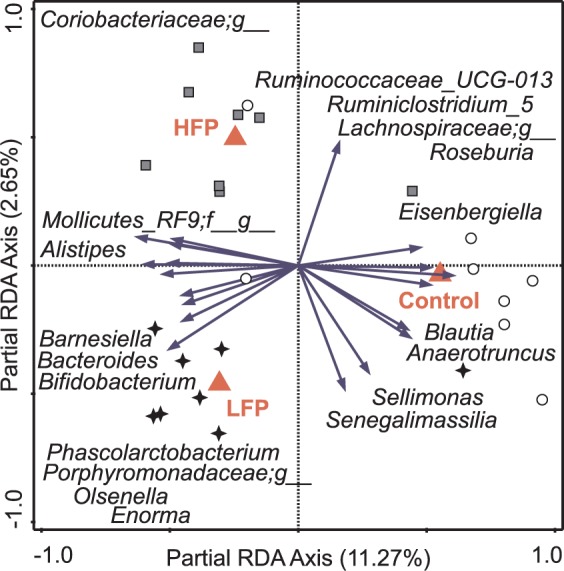
Triplot for partial redundancy analysis (RDA) of caecum microbiota composition of the high feather pecking (HFP) line at 2 weeks of age. Microbial groups for which the variation in relative abundance in the data is explained for at least 20% by the axes are represented as vectors. Nominal environmental treatment variables are represented by red triangles. Samples are grouped by treatment: HFP microbiota (grey squares), control (white circles) and low feather pecking (LFP) microbiota (black stars). Groups that could not be assigned to a specific genus are classified by the family name appended with “;g_”.

Newly hatched chicks received sterile saline (control), HFP or LFP microbiota transplantation within 6 h post-hatch to influence bacterial colonization^32^, and each day during the first 2 weeks post-hatch, a period when synapses are still being formed in the brain^33^. Microbiota was sampled from the luminal content of the ileum, caecum or colon at 5 days and 2 weeks of age to assess the effects of microbiota transplantation on recipients’ microbiota composition using 16S rRNA gene sequencing at the approximate genus-level. There was no overlap between microbiota composition of individual donors or the transplantation pools and that of recipients using a PCA (Sup. Fig. 1). Furthermore, multivariate redundancy analysis (RDA) showed a high overlap of line * treatment groups (Sup. Fig. 2), treatments (Sup. Fig. 3) and treatments within lines (Sup. Fig. 4–6). However, birds receiving the control treatment could be distinguished from birds receiving microbiota transplantation based on caecal microbial composition at 5 days and 2 weeks of age, where the control treatment explained 5.5% and 6.3%, respectively of the observed variation in microbiota composition (P = 0.032 and P = 0.004, respectively). Furthermore, within the HFP line, HFP birds receiving the control treatment could be distinguished from other groups in caecal microbial composition at 2 weeks of age, where the control treatment explained 11.2% of the observed variation in microbiota composition (P = 0.036; Fig. 2). This finding suggests the microbiota composition of HFP birds receiving the control treatment was distinct from that of HFP birds receiving HFP or LFP microbiota. To further analyse differences in microbiota composition, we determined whether line * treatment groups, treatments or treatments within lines differed in relative abundances of microbial groups. However, only a few microbial groups differed between line * treatment groups, treatments or treatments within lines, and most microbial groups that differed had on average low relative abundances (<1%) (Sup. Table 1). Overall, these results suggest limited effects of early-life microbiota transplantation on recipients’ microbiota composition.

### Early-life microbiota transplantation influenced behavioural responses

Several behavioural tests were performed to assess fearfulness and the stress response^34,35^. During treatment, birds were tested in a novel object test at 3 days of age and a novel environment test at 1 week of age. After treatment, birds were tested in a second novel object test at 5 weeks of age, a tonic immobility test at 9 weeks of age, an open field test at 13 weeks of age and a manual restraint test at 15 weeks of age. Fearfulness and the stress response were measured given that anxiety-like behaviour and stress were influenced by gut microbiota in rodents^2,5^, and fearfulness and stress sensitivity are related to the development of FP^19^. Here, findings with P-values < 0.1 were reported concerning the effects of line * treatment interactions, treatment and treatment within lines, but a complete overview is given in Sup. Table 2.

**Figure 3:**
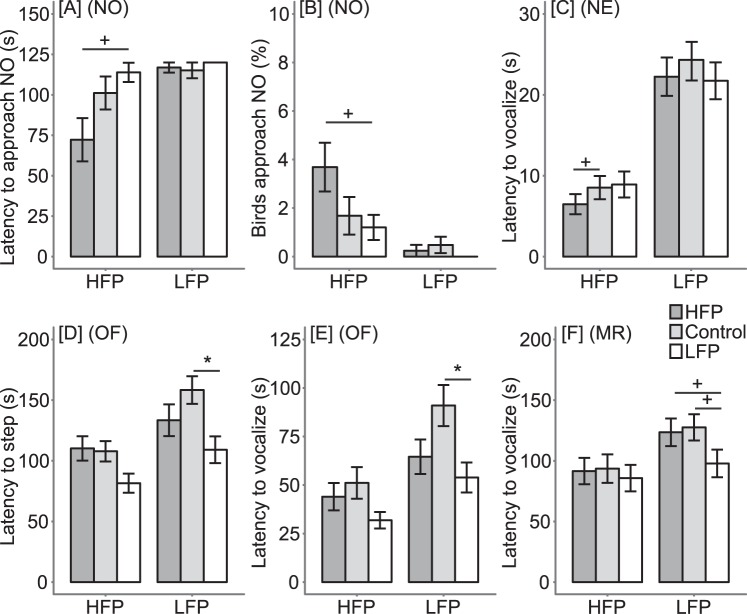
(**A**) Mean latency (±SE) for three birds to approach the novel object (NO) and (**B**) mean percentage (±SE) of birds approaching the NO at 3 days of age; (**C**) mean latency to vocalize (±SE) in the novel environment (NE) test at 1 week of age; (**D**) mean latency to step (±SE) and (**E**) mean latency to vocalize (±SE) in the open field (OF) test at 13 weeks of age; (**F**) mean latency to vocalize (±SE) in the manual restraint (MR) test at 15 weeks of age for the high (HFP) and low feather pecking (LFP) lines treated with HFP microbiota, control treatment or LFP microbiota. ^+^denotes tendencies (P < 0.1) and *denotes significant differences (P < 0.05) between treatments within lines.

During treatment, significant line * treatment interactions were found for latency to approach the novel object (χ^2^ = 16.32, df = 5, P = 0.006), the percentage of birds approaching the novel object (χ^2^ = 22.69, df = 5, P < 0.001) (Sup. Fig. 7) and flight attempts during the novel environment test (F_2,41_ = 3.27, P = 0.048) (Sup. Fig. 8). However, no treatment effects were found on behavioural responses to the novel object or novel environment. We further analysed the effect of treatment within lines because we were interested in whether treatments within a line differed from each other. Interestingly, we found tendencies for treatment effects within the HFP line, where it tended to affect the latency to approach the novel object (χ^2^ = 5.71, df = 2, P = 0.058; Fig. 3A), percentage of birds approaching the novel object (χ^2^ = 5.17, df = 2, P = 0.075; Fig. 3B) and the latency to vocalize in the novel environment test (F_2,20_ = 2.63, P = 0.097; Fig. 3C). HFP chicks receiving HFP microbiota tended to approach the novel object sooner (P = 0.064) and more birds tended to approach it (P = 0.091) compared to HFP chicks receiving LFP microbiota. Furthermore, HFP chicks receiving HFP microbiota tended to vocalize sooner compared to HFP chicks receiving the control treatment (P = 0.091). These results suggest that during treatment, HFP chicks receiving HFP microbiota showed more active behavioural responses compared to HFP chicks receiving LFP microbiota or the control treatment.

After treatment, significant line * treatment interactions were found on latency to approach the novel object (χ^2^ = 20.38, df = 5, P = 0.001) and percentage of birds approaching the novel object (χ^2^ = 19.35, df = 5, P = 0.002) (Sup. Fig. 7). Furthermore, treatment effects were found for the number of inductions needed to reach tonic immobility (F_2,43_ = 3.39, P = 0.043) and latency to step (F_2,43_ = 7.42, P = 0.002) and vocalize (F_2,43_ = 5.66, P = 0.007) in the open field test. Birds receiving LFP microbiota needed fewer inductions to reach tonic immobility compared to birds receiving HFP microbiota (P = 0.043) (Sup. Fig. 9A). In the open field test, birds receiving LFP microbiota stepped and vocalized sooner compared to birds receiving the control treatment (P = 0.001 and P = 0.005, respectively) and tended to step sooner compared to birds receiving HFP microbiota (P = 0.059) (Sup. Fig. 9B,C). To explore these treatment effects in more detail, we analysed treatment effects within lines. Interestingly, we found treatment effects within the LFP line, where it affected latency to step (F_2,20_ = 5.77, P = 0.011; Fig. 3D) and vocalize (F_2,20_ = 5.13, P = 0.016; Fig. 3E) in the open field test and latency to vocalize during restraint (F_2,20_ = 3.79, P = 0.04; Fig. 3F). In the open field test, LFP birds receiving LFP microbiota stepped (P = 0.008) and vocalized (P = 0.013) sooner compared to LFP birds receiving the control treatment. In the manual restraint test, LFP birds receiving LFP microbiota tended to vocalize sooner compared to LFP birds receiving HFP microbiota or the control treatment (P = 0.096 and P = 0.051, respectively). These results suggest that after treatment LFP birds receiving LFP microbiota showed more active behavioural responses compared to LFP birds receiving HFP microbiota or the control treatment.

In summary, these results indicate that behavioural responses were influenced by early-life microbiota transplantation, where effects were found during treatment in the HFP line and after treatment in the LFP line. Furthermore, in both lines, birds receiving homologous microbiota transplantation (i.e., receiving microbiota from their line) showed more active behavioural responses.

### Early-life microbiota transplantation influenced natural antibodies and peripheral serotonin, but not corticosterone

Several physiological characteristics were measured after the treatment period. Natural antibody titres (NAb, an antibody that binds antigen without prior intentional exposure to that antigen^36^) were measured at 5, 10 and 15 weeks of age as NAb’s play an essential role in both innate and adaptive immunity^37–39^ and were therefore used as a general immune characteristic. At 15 weeks of age, corticosterone level after manual restraint was used as an indicator of the physiological stress response^23^, and whole blood serotonin level was used as an indicator of central serotonin levels^40^. These physiological characteristics were measured given that the immune, stress and serotonergic systems are influenced by gut microbiota in rodents^3–5^, and these systems are related to FP^19–21^. Here, findings with P-values < 0.1 were reported concerning the effects of line * treatment interactions, treatment and treatment within lines, but a complete overview is given in Sup. Table 3.

**Figure 4:**
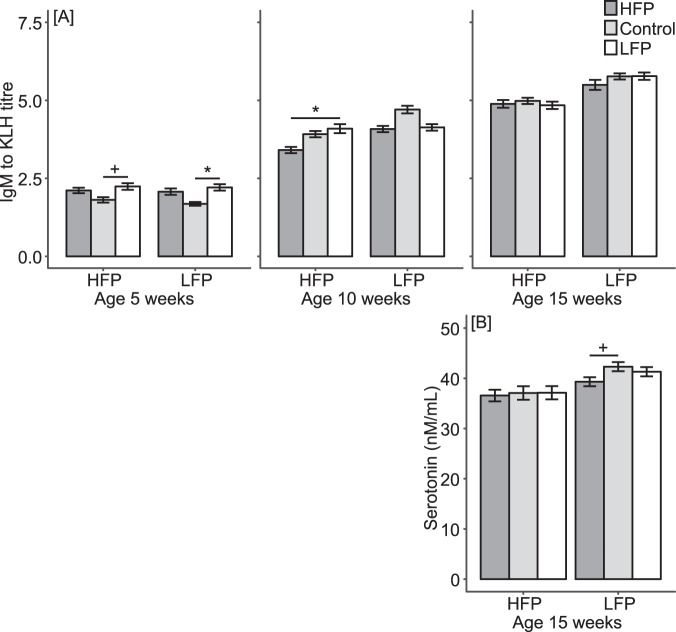
(**A**) Mean IgM natural antibody titres to keyhole limpet haemocyanin (KLH) (±SE) at 5, 10 and 15 weeks of age and (**B**) mean serotonin level (±SE) at 15 weeks of age for the high (HFP) and low feather pecking (LFP) lines treated with HFP microbiota, control treatment or LFP microbiota. ^+^denotes tendencies (P < 0.1) and *denotes significant differences (P < 0.05) between treatments within lines.

No effects of line * treatment interactions were found on any of the physiological characteristics. However, treatment effects were found on IgM NAb titres at 5 (F_2,43_ = 7.87, P = 0.001) and 10 weeks of age (F_2,43_ = 7.94, P = 0.031). Birds receiving the control treatment had lower IgM titres compared to birds receiving HFP (P = 0.018) or LFP microbiota (P = 0.001) at 5 weeks of age. However, at 10 weeks of age, they had higher IgM titres compared to birds receiving HFP microbiota (P = 0.026) (Sup. Fig. 10). To explore these treatment effects in more detail we analysed treatment effects within lines. In the HFP line, treatment tended to influence IgM NAb titres at 5 weeks of age (F_2,20_ = 3.18, P = 0.063) and significantly influenced IgM NAb titres at 10 weeks of age (F_2,20_ = 4.03, P = 0.034) (Fig. 4A). HFP birds receiving LFP microbiota tended to have higher IgM titres compared to HFP birds receiving the control treatment at 5 weeks of age (P = 0.064) and further had higher IgM titres compared to HFP birds receiving HFP microbiota at 10 weeks of age (P = 0.031). In the LFP line, we found a treatment effect on IgM NAb titres at 5 weeks of age (F_2,20_ = 4.41, P = 0.026) (Fig. 4A). LFP birds receiving LFP microbiota had higher IgM titres compared to LFP birds receiving the control treatment at 5 weeks of age (P = 0.025). Furthermore, treatment tended to influence peripheral serotonin within the LFP line (F_2,20_ = 2.62, P = 0.098; Fig. 4B), where LFP birds receiving HFP microbiota tended to have lower serotonin levels compared to LFP birds receiving the control treatment (P = 0.084).

Overall, these results suggest that early-life microbiota transplantation influenced IgM NAb in both lines and tended to influence peripheral serotonin in the LFP line after the treatment period. However, early-life microbiota transplantation did not influence IgG NAb or corticosterone.

### Early-life microbiota transplantation had limited effects on feather pecking

Feather pecking was observed between 0–1, 2–3, 4–5, 9–10 and 14–15 weeks of age at the pen level and was categorized into gentle FP (subdivided into exploratory and stereotyped FP) and severe FP. Here, gentle FP typically does not result in damage, and severe FP is the problematic behaviour in terms of damage to the recipient in animal production systems^19^. During and after the treatment period, significant line * treatment interactions were found for stereotyped FP and severe FP but not exploratory FP (for a complete overview see Sup. Table 4 and Sup. Fig. 11A–C). After treatment, a tendency for a treatment effect on exploratory FP was found for weeks 2–3 (F_2,43_ = 2.34, P = 0.083), where birds receiving HFP microbiota tended to show less exploratory FP compared to birds receiving LFP microbiota (P = 0.085). We explored treatment effects in more detail by analysing treatment effects within lines. In the LFP line, a tendency for a treatment effect was found for exploratory FP in weeks 4–5 (χ^2^ = 5.16, df = 2, P = 0.076). LFP birds receiving HFP microbiota tended to show more exploratory FP compared to LFP birds receiving the control treatment (P = 0.09). In summary, these results indicate that FP up to 15 weeks of age was not influenced by early-life microbiota transplantation.

## Discussion

This study aimed to identify effects of early-life microbiota transplantation on feather pecking (FP), and behavioural and physiological characteristics related to FP in high FP (HFP) and low FP (LFP) selection lines. We hypothesized that microbiota transplantation would result in recipients adopting a similar behavioural profile as that seen in the donor line. To summarize, behavioural responses were influenced by early-life microbiota transplantation, where the effects of homologous transplantation (i.e., receiving microbiota from their line) were seen during treatment in the HFP line and after treatment in the LFP line. Concerning physiological characteristics, we found effects on IgM natural antibodies in both lines and a tendency on peripheral serotonin in the LFP line, but not on IgG natural antibodies or corticosterone. Furthermore, early-life microbiota transplantation had limited effects on microbiota composition and FP.

### Microbiota composition of recipients

Early-life microbiota transplantation had limited effects on microbiota composition of recipients. A potential explanation for this could be that transplantation pools consisted of adult microbiota. Adult microbiota might not be able to colonize and remain within the gut of newly hatched chicks since the gut microbiota is still developing and undergoes a rapid succession until it is completely developed and stable around seven weeks of age^32,41^. Furthermore, we sampled luminal content instead of mucosal scrapings. Mucosa-associated microbiota composition might be more involved in communication with the host given its proximity^42^ and differs from luminal microbiota composition^43,44^. Mucosa-associated microbiota composition might have been altered by our microbiota transplantation, but the FP selection lines did not differ in mucosa-associated microbiota composition in our previous study^31^. Previous studies in rodents and quails found effects of microbiota transplantation on microbiota composition of recipients^13,17,45–47^. However, these studies used pseudo-germ-free (i.e., received antibiotic treatment before transplantation) or germ-free animals and most identified microbiota composition from faecal samples. Using (pseudo-) germ-free animals allows for stronger effects of microbiota transplantation on microbiota composition as these animals are sterile or depleted of microbiota and are often housed in a sterile environment. However, such animal models are rather extreme, making it difficult to translate findings to ‘normally’ occurring situations. Furthermore, identifying microbiota composition from different gut sections (i.e., ileum, caecum or colon) is crucial, as microbiota composition of faecal samples is variable because it originates from different gut sections^48^, which differ in microbiota composition^43,49,50^. Thus, differences found in previous studies might be due to faecal sampling.

Although limited effects of early-life microbiota transplantation on microbiota composition were found, microbiota transplantation did affect behavioural responses and natural antibodies and tended to affect peripheral serotonin. Early-life microbiota transplantation potentially influenced brain, immune and serotonergic system functioning. Microbiota transplantation was given during the first two weeks post hatch when both the brain and immune system are still developing^33,51,52^. There is extensive evidence that gut microbiota affects brain, central serotonergic and immune system functioning^3,4,53^, and it seems to influence peripheral serotonergic system functioning as well. For example, germ-free mice had lower mRNA levels of tryptophan hydroxylase (enzyme for serotonin synthesis) and higher mRNA levels of the serotonin transporter in intestinal cells^54^. However, it should be noted that we cannot exclude that other factors in the transplantation pools might have contributed to altering behavioural responses, natural antibodies and peripheral serotonin, for example, viruses or fungi^55,56^. We suggest that microbiota transplantation influenced brain, serotonergic and immune system functioning, which (in)directly resulted in differences in behavioural responses, natural antibodies and serotonin.

### Are recipients adopting the behavioural profile of donors?

Previously, HFP birds showed more active responses compared to LFP birds in the behavioural tests that were performed in the present study^24,25,27^ and other behavioural tests^57^. Thus, during the treatment period, behavioural responses seem to be adopted from donors in the HFP line as HFP chicks receiving HFP microbiota tended to show more active responses (i.e., approached novel object sooner and more birds approached it, vocalized sooner) compared to HFP chicks receiving LFP microbiota or the control treatment. In contrast, after the treatment period behavioural responses were not adopted from donors in the LFP line as LFP birds receiving LFP microbiota showed more active responses (i.e., stepped and vocalized sooner) compared to LFP birds receiving HFP microbiota or the control treatment. Previous studies in rodents show that behavioural profiles of donors are adopted by recipients via microbiota transplantation^13,45,47,58^. However, similar to our findings, a recent study using early-life microbiota transplantation in quails showed that birds adopted the behaviour of donors early in life, but that this effect was reversed later in life^17^. Thus, early-life microbiota transplantation can affect behavioural responses in poultry, and behavioural profiles can be adopted. However, these effects are dependent on age and genotype.

Although behavioural responses were influenced by early-life microbiota transplantation, FP was not, and recipients did not adopt FP behaviour of donors. An explanation for this finding might be that FP usually increases from the egg-laying period onwards (approximately 20 weeks of age)^59,60^, and we observed FP till 15 weeks of age. It should further be noted that effects on FP might be missed as FP was observed for a limited amount of time, and some variation in severe FP was observed between pens, which is probably caused by severe FP only being performed by few individuals^59^. Further research is needed to identify the potential effects of early-life microbiota transplantation on FP at adult age.

### Homologous microbiota transplantation

Interestingly, in both the HFP and LFP lines, homologous microbiota transplantation (i.e., receiving microbiota from their line) resulted in birds showing more active responses, which suggests reduced fearfulness given that silence and inactivity are related to high fearfulness^34,61^. Therefore, homologous transplantation could be a potential approach to lower fearfulness in chickens. Many studies show that FP is related to high fearfulness^62–64^, indicating that receiving homologous transplantation might reduce the risk of birds developing FP. However, it should be noted that no treatment effects within lines were found on severe FP or tonic immobility duration, the measure for innate fearfulness in chickens^34^. Furthermore, we previously showed that HFP birds had shorter tonic immobility duration compared to LFP birds^25^, suggesting that FP is related to low fearfulness in the FP selection lines. However, another study reported no difference in tonic immobility duration between the FP selection lines^65^. Thus, homologous transplantation could be used to reduce fearfulness in poultry, for example by providing faeces from mother hens to chicks, thereby potentially reducing the development of FP.

Microbiota transplantation might be seen as a type of vertical transmission, where microbiota is transferred from mother hens to chicks. Vertical transmission might play an important role in initiating a host-specific gut microbiota, which could improve the host’s immune system and brain development. For example, germ-free mice colonized with human microbiota showed impaired immune system development compared to mice colonized with mouse microbiota^66^, suggesting that host-specific microbiota is required for immune system maturation. Although this result is noted from a comparison of inter vs. intra species microbiota transplantation, it could still point to improved immune system development through homologous transplantation. Moreover, germ-free mice receiving homologous transplantation had higher brain-derived neurotrophic factor (BDNF) levels in the hippocampus but not in the amygdala compared to germ-free mice receiving microbiota from another strain^13^. This finding suggests that homologous transplantation might improve brain development, since BDNF is involved in neuronal differentiation, synapse formation and plasticity^67^. Thus, receiving homologous transplantation could improve the immune system and brain development, potentially altering natural antibodies and behavioural responses. Furthermore, fearfulness was shown to decrease with age in chickens^68,69^, suggesting that homologous transplantation might accelerate behavioural development. Further research is needed to identify whether homologous transplantation improves immune system and brain development in poultry.

### Effects of microbiota transplantation depend on the recipient’s genotype

During treatment, microbiota transplantation tended to influence behavioural responses in the HFP line. However, after treatment, it influenced behavioural responses in the LFP line. A potential explanation for this could be that the HFP line seems to have a more responsive immune system, which reacts more strongly to the environment^26–28^. A more responsive immune system might result in HFP birds responding more strongly to microbiota transplantation with the synthesis and release of pro-inflammatory cytokines^70^. Peripherally produced pro-inflammatory cytokines can act on the brain^71^, where they alter serotonergic and dopaminergic neurotransmission^72^, which have been indicated to play a role in the development of FP^20^. However, we did not identify pro-inflammatory cytokine levels or brain neurotransmission in the present study. Yet, direct-fed microbials were shown to alter intestinal mRNA levels of pro-inflammatory cytokines^73^, and probiotic treatment altered serotonergic and dopaminergic neurotransmission^16^ in broilers, indicating that microbiota could influence cytokine levels, and serotonergic and dopaminergic neurotransmission in poultry. Thus, further research is needed to identify the effects of microbiota transplantation on cytokine levels and brain neurotransmission in poultry. Furthermore, it should be noted that after the treatment period, both lines responded similarly to microbiota transplantation concerning IgM natural antibodies, and no differences were found for IgG natural antibodies. In addition, behavioural responses of HFP birds tended to be influenced by receiving HFP microbiota compared to LFP microbiota or the control treatment, indicating that receiving any type of adult microbiota composition was not sufficient to alter behavioural responses in the HFP line. It is possible that HFP microbiota had specific effects on other immune characteristics; however, this hypothesis needs further investigation.

After treatment, microbiota transplantation influenced behavioural responses in the LFP line. These effects do not seem to be explained by differences in peripheral serotonin. LFP birds receiving HFP microbiota tended to have lower peripheral serotonin levels compared to LFP birds receiving the control treatment, while behavioural differences were observed between LFP birds receiving LFP microbiota and LFP birds receiving HFP microbiota or the control treatment. Moreover, it should be noted that serotonin cannot cross the blood-brain barrier^74^; thus, caution is needed when using peripheral serotonin levels as an indicator for central serotonin levels. Nevertheless, it is interesting that LFP birds receiving HFP microbiota tended to have lower peripheral serotonin, as we previously found that HFP birds had lower peripheral serotonin compared to LFP birds^27^. This finding might point to an increased risk for developing FP in LFP birds receiving HFP microbiota as low peripheral serotonin levels are related to high FP^40,64,75^. However, the potential pathway through which microbiota transplantation influences behavioural responses in LFP birds remains unclear.

### Conclusion

This is the first study to investigate effects of early-life microbiota transplantation on FP, and behavioural and physiological characteristics related to FP. In conclusion, early-life microbiota transplantation influenced behavioural responses that are related to FP. Effects were seen during treatment in the HFP line and after treatment in the LFP line. In both lines, homologous microbiota transplantation resulted in more active behavioural responses, indicating lower fearfulness. Early-life microbiota transplantation further influenced physiological characteristics after treatment, including immune characteristics (natural antibodies) in both lines and peripheral serotonin in the LFP line, but had limited effects on microbiota composition, the physiological stress response (corticosterone) and FP. Thus, early-life microbiota transplantation had immediate and long-term effects on behavioural responses and long-term effects on immune characteristics and peripheral serotonin. However, the effects were genotype dependent. Since microbiota transplantation influenced behavioural and physiological characteristics that are related to FP, it could thereby influence the development of FP. However, microbiota transplantation did not influence FP up to adolescent age and more research is needed to identify whether it could influence FP later in life.

## Material and Methods

### Animals and housing

White Leghorn birds from the 19^th^ generation of lines selected for high (HFP) and low feather pecking (LFP) were used (see Kjaer *et al*.^76^ for the selection procedure). A total of 576 birds (HFP = 288 and LFP = 288) were hatched from two batches of eggs with 3 weeks between batches. Eggs were incubated at an average eggshell temperature of 37.8 °C and an average relative humidity of 55.7%. Eggs were not disinfected, and lines were distributed randomly within the incubator. Eggs were placed in hatching baskets on embryonic day 18, and cardboard was placed on the bottom to prevent cross-contamination. From embryonic day 19, we collected hatched chicks (dry and wet chicks) every 6 h. There was no light in the incubator, and eggshells were removed to limit chicks from pecking at the environment or eggshells through which they could obtain bacteria. Chicks received a neck tag with a unique number and their first treatment and were weighed. Chicks were then placed in separate hatching baskets according to line * treatment group (line [HFP or LFP] * treatment [HFP microbiota, control or LFP microbiota]) with 6 experimental groups in total, which were distributed randomly within the incubator. On embryonic day 21, chicks were sexed and placed in pens according to experimental group with an approximate 50/50 male/female distribution. Non-beak-trimmed birds were used and were housed in groups of 12 birds per pen. At 5 days, 2 weeks and 10 weeks of age, group size was reduced for microbiota sampling (n = 10–11 birds per pen, n = 7–10 birds per pen and n = 6–9 birds per pen, respectively). Batches had the same housing conditions and experimental setup with 4 pens for each experimental group and 24 pens in total, with an overall total of 8 pens for each experimental group and 48 pens for both batches. During hatching and for the first 5 weeks of age, extra hygienic measures were taken to prevent cross-contamination. Gloves were worn when handling birds and switched between treatments. Cover shoes were used when entering a pen and switched between pens.

At all times, water and feed were provided *ad libitum*. Birds received a commercial rearing diet 1 from hatching until 8 weeks of age and a commercial rearing diet 2 from 8 until 16 weeks of age for laying hens (Agruniek Rijnvallei Voer B.V.). Each floor pen (h: 2 m, l: 1 m, w: 2 m) had wood shavings on the floor, two perches installed 45 cm above the floor and visual barriers of 1.5 m high to prevent birds in adjacent pens from seeing each other. Post hatch, the temperature was maintained at approximately 33 °C and gradually lowered to 24 °C at 4 weeks of age. The light regime was 23 L:1D post-hatch and was gradually reduced to 8 L:16D at 4 weeks of age. Light intensity for each pen ranged between 45 and 81 LUX (average 62.6 LUX) as measured with a Voltcraft MS-1300 light meter (Conrad Electric Benelux). The study was originally designed to end at 30 weeks of age. However, due to unforeseen practical issues that would have seriously influenced our experimental results and were beyond our control, we had to postpone the start of the experiment and were subsequently unable to maintain birds for longer than 15 weeks of age. The experiment was approved by the Central Authority for Scientific Procedures on Animals according to Dutch law (no: AVD104002015150-1).

### Treatment

**Figure 5:**
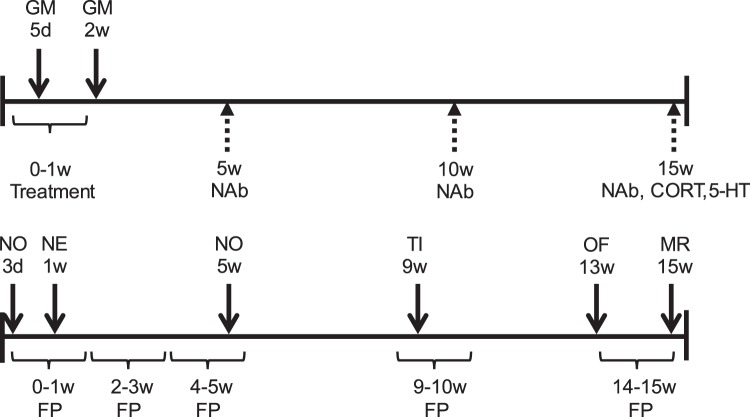
Timeline of the experiment. The upper line indicates physiological measures: microbiota sampling (above the line) and microbiota transplantation treatment and blood sampling (below the line) performed at specific ages in days (d) or weeks (w). GM = gut microbiota, NAb = natural antibodies, CORT = corticosterone and 5-HT = serotonin. The lower line indicates behavioural measures: behavioural tests (above the line) and feather pecking observations (below the line). FP = feather pecking observations, NO = novel object test, NE = novel environment test, TI = tonic immobility test, OF = open field test and MR = manual restraint test.

Microbiota transplantation consisted of a mixed pool of luminal content of the ileum, caeca and colon from either HFP or LFP adult birds collected during a previous experiment^31^. Pooled samples were stored at −80 °C until use. The number of viable aerobic and anaerobic microorganisms in pooled samples was determined using plate cultures with a blood agar medium. Plates were incubated overnight under aerobic or anaerobic conditions at 37 °C, and colonies were counted. Before treatment, pooled samples were defrosted in a 37 °C water bath for 5 min and then centrifuged at 5250 × g for 10 min. The microbial pellet was re-suspended in sterile saline (0.9% NaCl, half of the original volume was added). The control treatment consisted of sterile saline. Treatments were kept on ice in between processing steps and during administration. The first treatment was given within 6 h post-hatch. Thereafter, chicks received treatments daily during the first two weeks post-hatch. Each treatment consisted of 100 µL of the treatment solution administered orally using a pipette (see Fig. 5 for the timeline of the experiment).

### Microbiota sampling

At 5 days and 2 weeks of age, 8 birds of each experimental group (1 per pen) were randomly selected and sacrificed for the collection of gut microbiota. We collected luminal content from a ±2 cm midsection of the ileum (between Meckel’s diverticulum and the ileo-caeca-colic junction), one of the caeca and the colon. Samples were stored in cryovials at −80 °C until further analysis.

### Microbiota analysis

Microbiota composition of transplantation pools (HFP 5 replicates and LFP 5 replicates) and luminal content (n = 8 for each experimental group, gut section and age, total of n = 288) was determined via total DNA extraction, PCR amplification and sequencing as described previously^31^. PCR amplification was performed with primers directed to the V5-V6 region of the bacterial 16S rRNA gene, namely, BSF784F (5′-RGGATTAGATACCC) and 1064 R (5′-CGACRRCCATGCANCACCT). Data were processed using NG-Tax, an in-house bioinformatics pipeline, as described by Ramiro-Garcia *et al*.^77^, which resulted in a minimum of 29324 reads and a maximum of 607793 reads per sample.

### Behavioural observations and tests

Feather pecking (FP) behaviour was observed between 0–1, 2–3, 4–5, 9–10 and 14–15 weeks of age. Birds were further subjected to five behavioural tests: novel object, novel environment, open field, tonic immobility and manual restraint. The order of testing was randomized at an individual level, except for FP observations and novel object tests, which were randomized at the pen level because we observed FP and behavioural responses to the novel object at pen level. The experimenters were blinded to the lines and treatments.

### Feather pecking observations

Each pen was observed for 30 min, either in the morning (8:30 h-12:30 h) or in the afternoon (12:30 h-16:30 h) after a 2.5-min habituation period. FP was divided into gentle feather pecks (subdivided into exploratory FP and bouts of stereotyped FP) and severe FP as adapted from Newberry *et al*.^59^. Reliability between the two observers (inter-observer agreement) was high for all FP behaviours (Pearson correlations: exploratory FP (0.92), stereotyped FP (0.85) and severe FP (0.97)).

### Novel object test

At 3 days and 5 weeks of age, the response to a novel object (NO) was tested at the pen level. At 3 days of age (n = 48), the NO was a wooden block (h: 8 cm, l: 5 cm, w- 2.5 cm) wrapped with coloured tape (green, bright pink, light pink and yellow)^64^. At 5 weeks of age (n = 48), the NO test was repeated with a plastic stick (l: 50 cm, d: 3.5 cm) wrapped with coloured tape (red, white, green, black, and yellow)^78^. The test started 10 sec after one experimenter had placed the NO on the floor in the centre of the home pen. The latency for three birds to approach the NO at a distance of <25 cm and the number of birds that were within <25 cm of the NO were recorded by another experimenter every 10 sec for the 2-min test duration. Two experimenters tested all pens at 3 days and 5 weeks of age.

### Novel environment test

At 1 week of age, the response to a novel environment^64^ was tested for a duration of 1 min (n = 520). All birds from a pen were taken and placed in a cardboard box in front of the home pen. The average time difference between the first and last bird to be tested was 9 min. Birds were then individually taken to one of three test locations, where birds were placed inside a white bucket (h: 57 cm, l: 32 cm, w: 22 cm) at the start of the test. The latency to vocalize, the number of vocalizations and flight attempts were recorded. Together, three experimenters tested all birds where each experimenter tested approximately one-third of the birds.

### Tonic immobility test

At 9 weeks of age, birds were tested in a tonic immobility (TI) test^79^ for a maximum duration of 5 min (n = 458). Half of the birds in a pen were taken and transported in a cardboard box to a room near the testing rooms. The average time difference between the first and last bird to be tested was 8 min. Birds were individually taken to one of two test rooms, where they were placed in a supine position in a metal cradle with their head suspended from the side of the cradle. Each bird was restrained for 10 sec and when the bird remained in this position after release, TI duration was recorded until the bird returned to an upright position. If this occurred within 10 sec after release TI was induced again, with a maximum of three attempts at inducing TI. Together, two experimenters tested all birds, and each experimenter tested approximately half of the birds.

### Open field test

At 13 weeks of age, birds were tested in an open field (OF) test^80^ for a duration of 5 min (n = 409). Birds were individually taken and transported to the test room in a cardboard box. The OF was a square wooden enclosure (h: 1.22 m, l: 1.15 m, w: 1 m) with a video camera positioned above it. A bird was placed in the centre of the OF, and the test started when the lights were switched on. One experimenter recorded the latency to vocalize and the number of vocalizations. A second experimenter recorded the latency to step and the number of steps from a monitor in an adjacent room. Three experimenters tested all birds, and each experimenter tested approximately one-third of the birds for vocalizations or steps.

### Manual restraint test

At 15 weeks of age, birds were tested in a manual restraint (MR) test^81^ for a duration of 5 min (n = 409). Birds were individually taken and transported to one of two test rooms in a cardboard box. Birds were placed on their right side on a table. The right hand of the experimenter covered the bird’s back, and the left hand gently stretched the bird’s legs. The latencies to vocalize and struggle and the number of vocalizations and struggles were recorded. Together, four experimenters tested all birds, and each experimenter tested approximately one-fourth of the birds. Fifteen min after the start of MR, blood samples were drawn from the wing vein for assessment of the peak plasma corticosterone level^82^.

### Blood collection and analyses

Blood was collected from all birds at 5, 10 and 15 weeks of age. Blood was taken from the wing vein using a heparinized syringe and kept on ice after blood sampling. Blood samples for corticosterone and natural antibodies were centrifuged at 5250 × g for 10 min at room temperature, and the obtained plasma was stored at −20 °C until further analysis. Whole blood samples (1 mL) for the determination of serotonin were stored at −20 °C until further analysis^27^.

### Plasma IgM and IgG natural antibody titres

Samples from all weeks were used for the determination of IgM and IgG natural antibody titres against keyhole limpet haemocyanin via an indirect enzyme-linked immunosorbent assay as described previously^83^ with the following modifications. Serial dilutions of plasma were made in four steps starting with a 1:40,000 dilution in phosphate buffer saline containing 0.05% Tween 20 and 1% horse plasma (100 µL in each well). Peroxidase-conjugated goat-anti-chicken IgM (A30-102P, Bethyl; dilution 1:20,000) or goat-anti-chicken IgG (A30-104P, Bethyl; dilution 1:20,000) was used as secondary antibody (100 µL in each well).

### Plasma corticosterone

Samples from week 15 were used for the determination of plasma corticosterone concentrations via a radioimmunoassay kit (MP Biomedicals) as described previously^84^.

### Whole blood serotonin

Samples from week 15 were used for the determination of whole blood serotonin concentration (nmol/mL) via a fluorescence assay as described previously^81^. The centrifugation steps were performed at 931 × g, and fluorescence was determined in a Perkin-Elmer 2000 Fluorescence spectrophotometer at 295 and 540 nm.

### Statistical analysis

A power analysis was performed for the main variable FP. Using an alpha of 0.05 and a power of 0.8, we calculated that with a mean of 10 FP bouts and a standard deviation of 5 we needed a sample size of 8 pens per experimental group (pen is the experimental unit as individuals within one pen can influence each other’s behaviour).

To plot the microbial composition of the transplantation pools, principal component analysis was used as implemented in CANOCO 5 software package (Biometris). The relative contribution of 259 genus-level phylogenetic groups identified by 16S rRNA gene sequencing was used as response variables. To relate changes in microbial composition to explanatory variables, redundancy analysis was used as implemented in the CANOCO 5 software package. Line * treatment interaction was introduced as a nominal variable, and we further tested treatment and treatment effects within lines separately. The relative contribution of 259 genus-level phylogenetic groups identified by 16S rRNA gene sequencing was used as response variables. Analyses were performed for each age (5 days and 2 weeks of age) and gut section (ileum, caecum or colon) separately. The Monte Carlo Permutation test (number of permutations 499) was applied to test for significance of the effect of line * treatment, treatment or treatment within lines on microbiota composition. Batch and sex were included as covariates. P-values were corrected using a Benjamini Hochberg correction^31^.

SAS Software version 9.4 was used for statistical analysis (SAS Institute). Linear mixed models for line * treatment effects were tested for each age separately and consisted of fixed effects of line * treatment, line, treatment, sex and batch and the random effect of pen within line and treatment. Linear mixed models for treatment effects within lines consisted of fixed effects of treatment, sex and batch and the random effect of pen within treatment. Linear mixed models for FP behaviours and behavioural responses to NO did not include the fixed effect of sex or a random effect as they were tested at the pen level. Test time (morning 8:00 h–12:30 h or afternoon 12:30 h–18:00 h) was added as a fixed effect for the TI, OF and MR tests. Experimenter was added as a fixed effect for the NE, TI, OF and MR tests. The model residuals were visually examined for normality. Variables were square-root transformed (i.e., latency to vocalize and frequency of vocalizations in the NE test; TI duration; latency to vocalize and step, step and vocalization frequency in the OF test; latency to struggle and vocalize in the MR test; corticosterone and serotonin) to obtain normality of model residuals. Generalized linear mixed models with a binary distribution were used to test the effects of line*treatment, line and treatment or treatment effects within lines for flight attempts in the NE test, the number of inductions needed to reach TI, and struggle and vocalization frequency in the MR test. A backward regression procedure was used when fixed effects (i.e., line * treatment, test time or experimenter) had P > 0.1. Post hoc pairwise comparisons were corrected by Tukey–Kramer adjustment. Kruskal Wallis tests were used to analyse line * treatment, treatment and treatment effects within lines for individual microbial groups, stereotyped FP, severe FP and behavioural responses to the NO test, and post hoc comparisons were made with the Dwass, Steel, Critchlow-Fligner method. All data are presented as (untransformed) mean ± standard error (SE)^25,27^.

The datasets generated and/or analysed during the current study are available from the corresponding author on reasonable request.

## Supplementary information

Supplementary information.
