# Supplementary material for: Early-life microbiota transplantation affects behavioural responses, serotonin and immune characteristics in chicken lines divergently selected on feather pecking
Source: Sci Rep. 2020 Feb 17;10:2750. doi: 10.1038/s41598-020-59125-w (PMC7026165; doi:10.1038/s41598-020-59125-w)
Supplement: Supplementary file 1 — Supplementary information. [file 41598_2020_59125_MOESM1_ESM.pdf]

Supplementary Information

Title

Early-life microbiota transplantation affects behavioural responses, serotonin and immune characteristics in chicken lines divergently selected on feather pecking

Authors

Jerine A.J. van der Eijk[1,2*], T. Bas Rodenburg[2,3], Hugo de Vries[4], Joergen B. Kjaer[5], Hauke Smidt[4], Marc Naguib[1], Bas Kemp[2] and Aart Lammers[2]

[1] Behavioural Ecology Group, Department of Animal Sciences, Wageningen University and Research, Wageningen, the Netherlands

[2] Adaptation Physiology Group, Department of Animal Sciences, Wageningen University and Research, Wageningen, the Netherlands

[3] Department of Animals in Science and Society, Faculty of Veterinary Medicine, Utrecht University, Utrecht, the Netherlands

[4] Laboratory of Microbiology, Wageningen University and Research, Wageningen, the Netherlands

[5] Friedrich-Loeffler-Institut, Institute of Animal Welfare and Animal Husbandry, Celle, Germany

*Corresponding author: jerine.vandereijk@wur.nl (J.A.J. van der Eijk)

Behavioural Ecology Group, Department of Animal Sciences, Wageningen University and Research, P.O. Box 338, 6700 AH Wageningen, the Netherlands

Table 1. Relative abundance (%) of microbial groups of the high (HFP) and low feather pecking (LFP) lines receiving HFP, control or LFP microbiota treatment as determined from luminal content of the ileum, caecum or colon at 5 days and 2 weeks of age.

**Line**	**HFP**			**LFP**			**P-values**				
**Treatment**	**HFP**	**Control**	**LFP**	**HFP**	**Control**	**LFP**	**Line * Treatment**	**Line**	**Treatment**	**Treatment within line HFP**	**Treatment within line LFP**
**Age (5 days)**											
Ileum	**n=8**	**n=8**	**n=8**	**n=8**	**n=8**	**n=8**					
Candidatus Arthromitus	0.0 ± 0.0	0.0 ± 0.0	0.0 ± 0.04	0.0 ± 0.0	0.0 ± 0.0	0.4 ± 0.18	< 0.01	ns	< 0.01	ns	< 0.01
Enterococcus	0.3 ± 0.10	3.9 ± 2.4	0.8 ± 0.38	0.6 ± 0.58	0.1 ± 0.05	0.3 ± 0.21	ns	< 0.01	ns	ns	ns
Caecum	**n=8**	**n=8**	**n=8**	**n=8**	**n=8**	**n=8**					
Anaerofilum	0.2 ± 0.09	0.0 ± 0.0	0.0 ± 0.0	0.2 ± 0.09	0.2 ± 0.12	0.0 ± 0.0	ns	ns	< 0.05	< 0.05	ns
Anaerotruncus	0.4 ± 0.14	0.1 ± 0.04	0.7 ± 0.57	0.5 ± 0.25	0.4 ± 0.28	0.7 ± 0.40	ns	ns	< 0.05	ns	ns
Coriobaceriaceae;g__	0.3 ± 0.35	0.0 ± 0.0	0.0 ± 0.02	0.3 ± 0.16	0.1 ± 0.08	0.0 ± 0.03	< 0.05	ns	< 0.01	< 0.05	ns
*Clostridiales vadinBB60 group;g_*	0.7 ± 0.43	0.3 ± 0.22	2.0 ± 1.0	0.7 ± 0.20	0.5 ± 0.34	0.8 ± 0.22	ns	ns	< 0.05	ns	ns
Lachnoclostridium	1.7 ± 0.38	0.2 ± 0.10	1.5 ± 0.61	1.3 ± 0.76	0.5 ± 0.28	1.0 ± 0.32	< 0.05	ns	< 0.05	< 0.05	ns
Tyzzerella	0.3 ± 0.10	0.0 ± 0.01	0.1 ± 0.06	0.1 ± 0.07	0.1 ± 0.07	0.0 ± 0.01	ns	ns	ns	< 0.05	ns
Ruminococcus	0.5 ± 0.30	0.0 ± 0.0	0.0 ± 0.01	0.0 ± 0.04	0.0 ± 0.02	0.0 ± 0.0	< 0.05	ns	ns	< 0.05	ns
Ruminococcaceae;g__	0.7 ± 0.17	0.1 ± 0.06	0.9 ± 0.22	1.3 ± 0.43	0.5 ± 0.27	1.0 ± 0.38	< 0.05	ns	< 0.01	< 0.05	ns
Ruminiclostridium 9	0.3 ± 0.08	0.2 ± 0.07	1.0 ± 0.22	1.0 ± 0.58	0.9 ± 0.19	0.6 ± 0.16	< 0.01	< 0.05	ns	< 0.01	ns
Ruminococcaceae UCG 005	0.0 ± 0.04	0.0 ± 0.02	0.2 ± 0.07	0.2 ± 0.16	0.0 ± 0.03	0.1 ± 0.08	ns	ns	ns	< 0.05	ns
Lactobacillus	9.2 ± 1.90	13.9 ± 3.72	9.5 ± 3.06	18.9 ± 4.33	8.0 ± 1.47	6.2 ± 1.31	ns	ns	ns	ns	< 0.05
Lachnospiraceae FCS020 group	0.1 ± 0.08	0.0 ± 0.02	0.0 ± 0.05	0.0 ± 0.03	0.0 ± 0.00	0.2 ± 0.09	< 0.05	ns	< 0.05	ns	< 0.01
Klebsiella	0.0 ± 0.0	0.7 ± 0.54	0.2 ± 0.13	0.9 ± 0.94	1.9 ± 1.58	0.0 ± 0.00	ns	ns	< 0.05	ns	ns
Intestinimonas	0.1 ± 0.05	0.1 ± 0.07	0.3 ± 0.11	0.1 ± 0.06	0.1 ± 0.07	0.3 ± 0.11	ns	ns	< 0.05	ns	< 0.05
Colon	**n=6**	**n=8**	**n=8**	**n=8**	**n=8**	**n=8**					
Anaerofilum	0.0 ± 0.0	0.0 ± 0.0	0.0 ± 0.0	0.0 ± 0.2	0.2 ± 0.16	0.0 ± 0.02	ns	< 0.05	ns	ns	ns
Candidatus Arthromitus	0.0 ± 0.0	0.0 ± 0.0	0.1 ± 0.07	0.0 ± 0.0	0.0 ± 0.0	0.7 ± 0.32	< 0.01	ns	< 0.01	ns	< 0.01
Erysipelotrichaceae;g__	0.1 ± 0.06	0.0 ± 0.0	0.0 ± 0.0	0.0 ± 0.0	0.0 ± 0.0	0.6 ± 0.47	ns	< 0.05	ns	ns	ns

Table 1 (continued).

**Line**	**HFP**			**LFP**			**P-values**				
**Treatment**	**HFP**	**Control**	**LFP**	**HFP**	**Control**	**LFP**	**Line * Treatment**	**Line**	**Treatment**	**Treatment within line** HFP	**Treatment within line** LFP
**Age (2 weeks)**											
Ileum	**n=8**	**n=8**	**n=8**	**n=8**	**n=8**	**n=8**					
Peptostreptococcaceae;g__	1.7 ± 0.63	0.2 ± 0.13	2.1 ± 1.52	0.6 ± 0.20	0.1 ± 0.08	0.5 ± 0.34	< 0.05	ns	< 0.01	ns	< 0.05
Aeriscardovia	0.0 ± 0.03	0.0 ± 0.0	0.0 ± 0.0	0.4 ± 0.23	0.0 ± 0.02	0.5 ± 0.47	ns	< 0.05	ns	ns	ns
Streptococcus	0.2 ± 0.15	0.3 ± 0.31	0.4 ± 0.35	0.2 ± 0.18	0.0 ± 0.0	0.0 ± 0.02	ns	< 0.05	ns	ns	ns
Caecum	**n=8**	**n=8**	**n=8**	**n=8**	**n=8**	**n=8**					
Bifidobacterium	0.5 ± 0.13	0.1 ± 0.06	1.9 ± 0.74	1.8 ± 1.23	1.8 ± 1.25	0.5 ± 0.21	ns	ns	ns	< 0.05	ns
Enorma	0.2 ± 0.14	0.0 ± 0.0	0.2 ± 0.08	0.4 ± 0.24	0.0 ± 0.03	0.4 ± 0.22	< 0.01	ns	< 0.01	< 0.05	< 0.05
Coriobacteriaceae;g__	0.1 ± 0.04	0.6 ± 0.39	0.2 ± 0.12	0.9 ± 0.60	0.3 ± 0.12	0.3 ± 0.19	ns	ns	ns	< 0.05	ns
Porphyromonadaceae;g__	0.3 ± 0.16	0.0 ± 0.02	0.3 ± 0.08	0.2 ± 0.15	0.1 ± 0.05	0.3 ± 0.15	ns	ns	< 0.05	< 0.05	ns
Eisenbergiella	0.5 ± 0.11	2.4 ± 0.83	0.7 ± 0.20	0.6 ± 0.18	1.2 ± 0.29	1.1 ± 0.29	< 0.05	ns	< 0.01	< 0.05	ns
Barnesiella	0.2 ± 0.09	0.0 ± 0.02	0.3 ± 0.14	0.1 ± 0.06	0.0 ± 0.02	0.2 ± 0.10	ns	ns	< 0.05	ns	ns
Alistipes	2.3 ± 0.63	1.5 ± 1.23	2.0 ± 0.55	1.8 ± 0.93	0.7 ± 0.42	2.5 ± 0.75	ns	ns	< 0.05	ns	ns
Hydrogenoanaerobacterium	0.0 ± 0.0	0.1 ± 0.04	0.0 ± 0.01	0.0 ± 0.0	0.1 ± 0.05	0.0 ± 0.02	ns	ns	< 0.05	ns	ns
Roseburia	0.0 ± 0.0	0.3 ± 0.18	0.0 ± 0.0	0.1 ± 0.07	0.0 ± 0.0	0.0 ± 0.04	< 0.05	ns	ns	< 0.05	ns
Collinsella	0.0 ± 0.02	0.0 ± 0.0	0.0 ± 0.02	0.1 ± 0.03	0.0 ± 0.0	0.0 ± 0.01	ns	ns	< 0.05	ns	< 0.05
Slackia	0.2 ± 0.12	0.0 ± 0.03	0.3 ± 0.20	0.1 ± 0.04	0.0 ± 0.03	0.4 ± 0.18	ns	ns	< 0.05	ns	< 0.05
Christensenellaceae R 7 group	0.8 ± 0.33	0.4 ± 0.22	0.6 ± 0.20	0.1 ± 0.07	1.3 ± 1.11	0.7 ± 0.19	ns	ns	ns	ns	< 0.05
Faecalitalea	0.0 ± 0.0	0.0 ± 0.02	0.0 ± 0.0	0.5 ± 0.31	0.0 ± 0.0	0.0 ± 0.0	< 0.05	ns	ns	ns	< 0.05
Phascolarctobacterium	0.6 ± 0.26	0.5 ± 0.39	0.9 ± 0.30	1.3 ± 0.57	0.3 ± 0.19	1.0 ± 0.36	ns	ns	< 0.05	ns	ns
Clostridiales vadinBB60 group g0	0.1 ± 0.11	0.6 ± 0.27	0.1 ± 0.03	0.2 ± 0.08	0.7 ± 0.25	0.2 ± 0.10	ns	ns	< 0.05	ns	ns
Parabacteroides	0.2 ± 0.11	0.0 ± 0.0	0.1 ± 0.04	0.4 ± 0.21	0.1 ± 0.08	0.4 ± 0.13	ns	< 0.05	ns	ns	ns
Tyzzerella 3	0.0 ± 0.0	0.0 ± 0.02	0.0 ± 0.0	0.1 ± 0.08	0.2 ± 0.10	0.1 ± 0.11	ns	< 0.05	ns	ns	ns
Oscillospira	0.0 ± 0.0	0.0 ± 0.0	0.0 ± 0.0	0.0 ± 0.02	0.0 ± 0.02	0.1 ± 0.03	ns	< 0.01	ns	ns	ns
Subdoligranulum	3.3 ± 0.65	2.0 ± 0.52	4.3 ± 1.89	2.0 ± 0.84	2.3 ± 0.63	1.6 ± 0.35	ns	< 0.05	ns	ns	ns
Colon	**n=8**	**n=7**	**n=7**	**n=8**	**n=7**	**n=8**					
Bifidobacterium	1.1 ± 1.06	0.0 ± 0.0	0.4 ± 0.17	0.1 ± 0.06	0.2 ± 0.13	0.1 ± 0.03	ns	ns	ns	< 0.05	ns
Lactobacillus	52.2 ± 9.88	56.2 ± 13.12	58.6 ± 12.55	34.6 ± 10.65	82.2 ± 4.40	36.4 ± 9.93	ns	ns	ns	ns	< 0.05
Peptostreptococcaceae;g__	34.3 ± 8.26	11.5 ± 1.73	10.8 ± 4.80	30.5 ± 9.28	4.3 ± 1.70	20.1 ± 6.37	< 0.05	ns	< 0.05	ns	< 0.05

Values represent means ± SE; The genus-level groups with a relative abundance lower than 0.1% in all treatment groups are not shown; Microbial groups that could not be assigned to a specific genus are classified by the family name appended with ";g_".

Table 2. Responses to behavioural tests at different ages of the high (HFP) and low feather pecking (LFP) lines receiving HFP, control or LFP microbiota treatment.

**Line**	**HFP**			**LFP**			**P-values**				
										**Treatment within line**	
**Treatment**	**HFP**	**Control**	**LFP**	**HFP**	**Control**	**LFP**	**Line * Treatment**	**Line**	**Treatment**	**HFP**	**LFP**
**Novel object (3d)**	**n=8**	**n=8**	**n=8**	**n=8**	**n=8**	**n=8**					
Lat. to approach	72.3 ± 13.37	101.1 ± 10.23	113.9 ± 5.98	116.9 ± 3.1	115.1 ± 4.88	120.0 ± 0.0	< 0.01	< 0.01	= 0.1	< 0.1	ns
# birds approach	3.7 ± 1.00	1.7 ± 0.78	1.2 ± 0.52	0.2 ± 0.24	0.5 ± 0.34	0.0 ± 0.0	< 0.01	< 0.01	ns	< 0.1	ns
**Novel environment (1w)**	**n=86**	**n=88**	**n=87**	**n=85**	**n=87**	**n=87**					
Lat. to vocalize	6.5 ± 1.25	8.5 ± 1.43	9.0 ± 1.59	22.2 ± 2.38	24.3 ± 2.39	21.7 ± 2.28	ns	< 0.01	ns	< 0.1	ns
Vocalization freq.	1.2 ± 0.06	1.1 ± 0.06	1.1 ± 0.06	0.4 ± 0.04	0.3 ± 0.04	0.3 ± 0.04	ns	< 0.01	ns	ns	ns
Flight attempts	0.4 ± 0.10	0.6 ± 0.13	0.6 ± 0.11	0.3 ± 0.08	0.1 ± 0.05	0.3 ± 0.07	< 0.05	< 0.01	ns	ns	ns
**Novel object (5w)**	**n=8**	**n=8**	**n=8**	**n=8**	**n=8**	**n=8**					
Lat. to approach	84.6 ± 12.08	87.6 ± 9.69	78.1 ± 13.35	120.0 ± 0.0	112.1 ± 7.88	120.0 ± 0.0	< 0.01	< 0.01	ns	ns	ns
# birds approach	4.5 ± 1.29	2.1 ± 0.66	5.7 ± 1.64	0.5 ± 0.55	1.2 ± 0.87	0.5 ± 0.21	< 0.01	< 0.01	ns	ns	ns
**Tonic immobility (9w)**	**n=74**	**n=79**	**n=76**	**n=73**	**n=78**	**n=78**					
TI duration	142.4 ± 14.52	107.6 ± 11.89	106.9 ± 12.82	124.9 ± 14.32	116.6 ± 11.52	120.6 ± 12.64	ns	ns	ns	ns	ns
# inductions	1.6 ± 0.09	1.5 ± 0.08	1.4 ± 0.09	1.5 ± 0.09	1.3 ± 0.08	1.4 ± 0.08	ns	ns	< 0.05	ns	ns
**Open field (13w)**	**n=66**	**n=71**	**n=68**	**n=64**	**n=70**	**n=70**					
Lat. to step	110.2 ± 10.08	107.8 ± 8.44	82.7 ± 7.86	133.4 ± 13.09	158.3 ± 11.42	109.1 ± 11.0	ns	< 0.01	< 0.01	ns	= 0.01
Step freq.	14.8 ± 1.59	15.8 ± 1.38	16.8 ± 1.46	14.2 ± 1.78	11.7 ± 1.63	15.6 ± 1.81	ns	= 0.01	ns	ns	ns
Lat. to vocalize	44.0 ± 7.08	51.1 ± 8.14	33.6 ± 4.50	64.6 ± 8.87	91.0 ± 10.60	53.5 ± 7.62	ns	< 0.01	< 0.01	ns	< 0.05
Vocalization freq.	18.0 ± 1.51	18.6 ± 1.61	19.4 ± 1.51	14.3 ± 1.92	12.8 ± 1.92	15.9 ± 1.80	ns	< 0.01	ns	ns	ns
**Manual restraint (15w)**	**n=66**	**n=70**	**n=69**	**n=64**	**n=70**	**n=70**					
Lat. to struggle	201.9 ± 12.20	227.6 ± 10.56	202.9 ± 10.92	204.8 ± 11.43	212.9 ± 11.03	189.3 ± 11.11	ns	ns	< 0.1	ns	ns
Struggle freq.	0.8 ± 0.14	1.3 ± 0.48	0.8 ± 0.13	0.8 ± 0.12	0.5 ± 0.08	0.9 ± 0.17	ns	ns	ns	ns	ns
Lat. to vocalize	91.6 ± 10.84	93.6 ± 11.71	85.8 ± 10.95	123.5 ± 11.42	127.6 ± 10.79	97.9 ± 11.37	ns	< 0.01	ns	ns	< 0.05
Vocalization freq.	10.1 ± 1.22	11.1 ± 1.67	11.1 ± 1.08	8.2 ± 1.90	6.6 ± 1.22	7.7 ± 0.67	ns	ns	ns	ns	ns

Values represent means ± SE; Age in days (d) or weeks (w) between brackets; Lat: latency in seconds; #: number; Freq: frequency in number/min; TI duration: tonic immobility duration in seconds.

Table 3. Physiological characteristics at different ages of the high (HFP) and low feather pecking (LFP) lines receiving HFP, control or LFP microbiota treatment.

**Line**	**HFP**			**LFP**			**P-values**				
**Treatment**	**HFP**	**Control**	**LFP**	**HFP**	**Control**	**LFP**	**Line * Treatment**	**Line**	**Treatment**	**Treatment within line HFP**	**Treatment within line LFP**
**Natural antibody titres (5w)**	**n=74**	**n=79**	**n=76**	**n=73**	**n=79**	**n=78**					
IgM	2.1 ± 0.09	1.8 ± 0.09	2.2 ± 0.11	2.1 ± 0.10	1.7 ± 0.06	2.2 ± 0.10	ns	ns	< 0.01	< 0.1	< 0.05
IgG	1.6 ± 0.09	1.5 ± 0.07	1.6 ± 0.08	1.4 ± 0.10	1.5 ± 0.09	1.5 ± 0.10	ns	ns	ns	ns	ns
**Natural antibody titres (10w)**	**n=74**	**n=79**	**n=76**	**n=73**	**n=78**	**n=78**					
IgM	3.4 ± 0.10	3.9 ± 0.10	4.1 ± 0.14	4.1 ± 0.10	4.7 ± 0.12	4.1 ± 0.10	ns	< 0.01	< 0.05	< 0.05	< 0.1
IgG	3.3 ± 0.20	3.0 ± 0.13	3.2 ± 0.22	2.8 ± 0.17	2.9 ± 0.15	2.6 ± 0.12	ns	< 0.05	ns	ns	ns
**Natural antibody titres (15w)**	**n=66**	**n=70**	**n=68**	**n=65**	**n=70**	**n=70**					
IgM	4.9 ± 0.13	5.0 ± 0.10	4.8 ± 0.12	5.5 ± 0.16	5.8 ± 0.10	5.8 ± 0.12	ns	< 0.01	ns	ns	ns
IgG	4.5 ± 0.21	4.5 ± 0.21	4.3 ± 0.21	3.6 ± 0.19	4.0 ± 0.20	3.9 ± 0.20	ns	< 0.01	ns	ns	ns
**Corticosterone (15w)**	**n=66**	**n=70**	**n=68**	**n=64**	**n=70**	**n=69**					
Corticosterone (ng/mL)	4.9 ± 0.37	6.0 ± 0.51	6.9 ± 0.79	5.7 ± 0.36	6.2 ± 0.49	6.3 ± 0.64	ns	ns	ns	ns	ns
**Serotonin (15w)**	**n=66**	**n=69**	**n=68**	**n=62**	**n=70**	**n=69**					
Serotonin (nM/mL)	36.6 ± 1.15	37.1 ± 1.35	37.1 ± 1.32	39.3 ± 0.90	42.3 ± 0.90	41.3 ± 0.92	ns	< 0.01	ns	ns	< 0.1

Values represent means ± SE; Age in days (d) or weeks (w) between brackets.

Table 4. Feather pecking (FP) behaviour (exploratory FP, stereotyped FP (bouts) and severe FP) at different ages of the high (HFP) and low feather pecking (LFP) lines receiving HFP, control or LFP microbiota treatment (n = 8 per experimental group).

Line	HFP			LFP			P-values				
Treatment	HFP	Control	LFP	HFP	Control	LFP	Line * Treatment	Line	Treatment	Treatment within line HFP	Treatment within line LFP
**Age (0-1 weeks)**											
Exploratory FP	5.3 ± 0.49	5.8 ± 0.34	4.6 ± 0.58	2.4 ± 0.15	2.1 ± 0.22	2.3 ± 0.17	ns	< 0.01	ns	ns	ns
Stereotyped FP	0.4 ± 0.10	0.9 ± 0.37	0.6 ± 0.20	0.2 ± 0.07	0.1 ± 0.05	0.1 ± 0.04	< 0.05	< 0.01	ns	ns	ns
Severe FP	4.5 ± 3.28	4.1 ± 1.96	1.4 ± 0.33	1.4 ± 0.96	0.3 ± 0.07	0.5 ± 0.16	< 0.01	< 0.01	ns	ns	ns
**Age (2-3 weeks)**											
Exploratory FP	3.3 ± 0.53	4.8 ± 0.57	5.5 ± 0.83	2.5 ± 0.35	2.9 ± 0.61	2.8 ± 0.42	ns	< 0.01	< 0.1	< 0.1	ns
Stereotyped FP	0.7 ± 0.28	1.5 ± 0.38	1.2 ± 0.44	0.3 ± 0.14	0.4 ± 0.13	0.2 ± 0.11	< 0.05	< 0.01	ns	ns	ns
Severe FP	13.4 ± 7.21	31.8 ± 13.82	12.2 ± 4.13	3.0 ± 1.79	1.1 ± 0.82	2.2 ± 1.43	= 0.01	< 0.01	ns	ns	ns
**Age (4-5 weeks)**											
Exploratory FP	2.5 ± 0.41	3.3 ± 0.86	3.8 ± 0.56	3.3 ± 0.66	1.8 ± 0.47	2.7 ± 0.53	ns	ns	ns	ns	< 0.1
Stereotyped FP	1.2 ± 0.66	2.5 ± 1.53	1.2 ± 0.59	0.6 ± 0.29	0.2 ± 0.08	0.7 ± 0.38	ns	< 0.05	ns	ns	ns
Severe FP	6.3 ± 4.81	15.8 ± 6.64	25.5 ± 12.3	6.5 ± 4.60	0.7 ± 0.39	1.8 ± 0.88	< 0.05	< 0.01	ns	ns	ns
**Age (9-10 weeks)**											
Exploratory FP	3.1 ± 0.77	3.0 ± 0.86	2.0 ± 0.64	1.7 ± 0.26	1.4 ± 0.23	1.4 ± 0.11	ns	< 0.05	ns	ns	ns
Stereotyped FP	0.3 ± 0.09	0.5 ± 0.26	0.2 ± 0.06	0.2 ± 0.07	0.1 ± 0.03	0.1 ± 0.03	ns	< 0.05	ns	ns	ns
Severe FP	1.1 ± 0.52	6.6 ± 4.44	2.9 ± 2.29	1.0 ± 0.41	0.6 ± 0.16	0.5 ± 0.12	ns	ns	ns	ns	ns
**Age (14-15 weeks)**											
Exploratory FP	3.6 ± 0.47	2.5 ± 0.71	2.9 ± 0.74	1.6 ± 0.39	1.9 ± 0.41	1.1 ± 0.25	ns	< 0.01	ns	ns	ns
Stereotyped FP	0.6 ± 0.11	0.3 ± 0.13	0.8 ± 0.30	0.2 ± 0.06	0.2 ± 0.06	0.2 ± 0.08	< 0.1	< 0.05	ns	ns	ns
Severe FP	4.5 ± 0.63	6.4 ± 4.30	6.3 ± 2.11	1.2 ± 0.28	1.0 ± 0.34	0.5 ± 0.21	< 0.01	< 0.01	ns	ns	< 0.1

Values represent means ± SE; Average number of pecks or bouts per bird per hour (number of pecks or bouts per pen per hour divided by the number of birds in that pen).

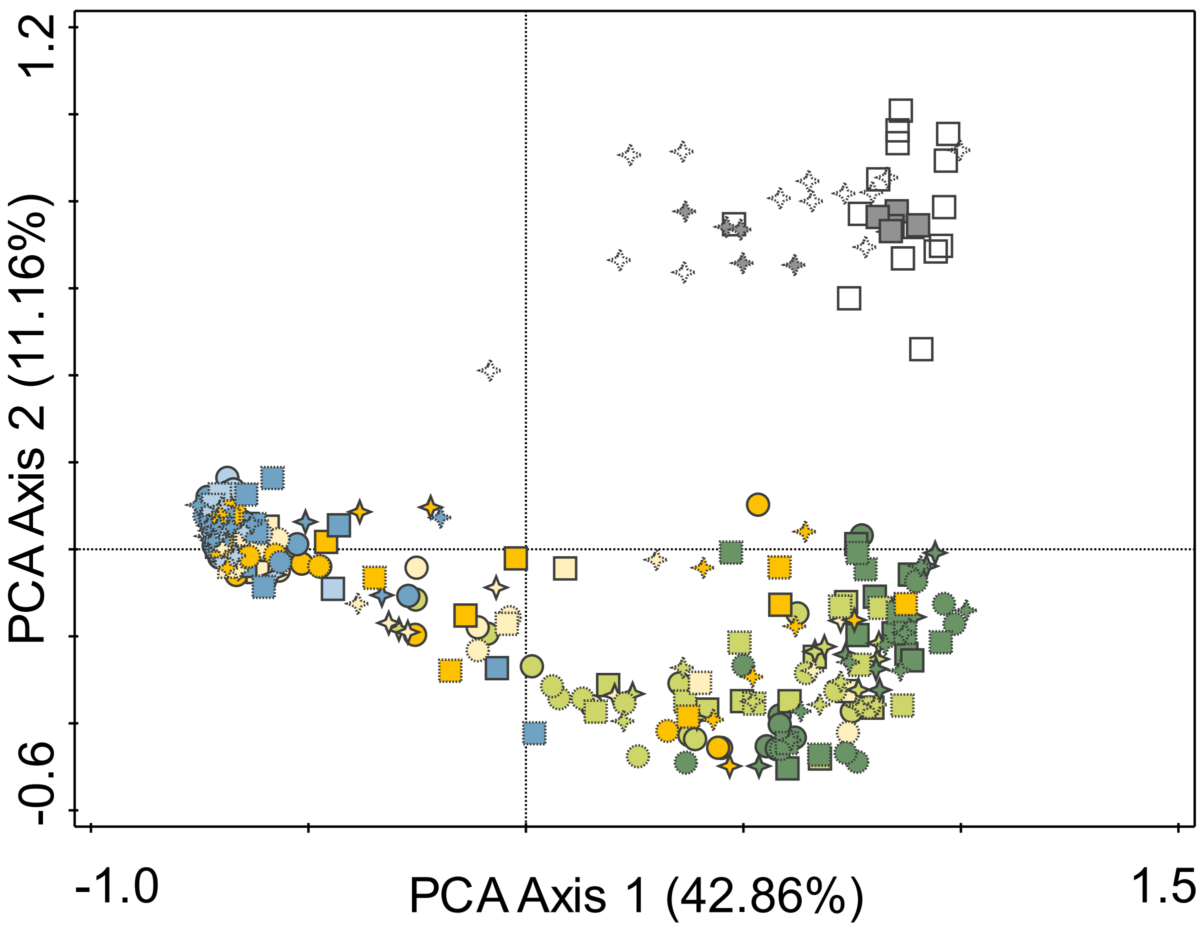

Figure 1. Biplot for principal component analysis (PCA) of donors', transplantation pools' and recipients' microbiota composition. Samples are grouped by line: high feather pecking (HFP; solid line) and low feather pecking (LFP; dotted line), by individual donors (open symbol) and transplantation pools (grey symbol), by treatment: HFP (squares), Control (circles) and LFP (stars), by gut section: ileum (blue), caecum (green) and colon (yellow), by age: 5 days (light blue, green or yellow) and 2 weeks of age (dark blue, green or yellow).

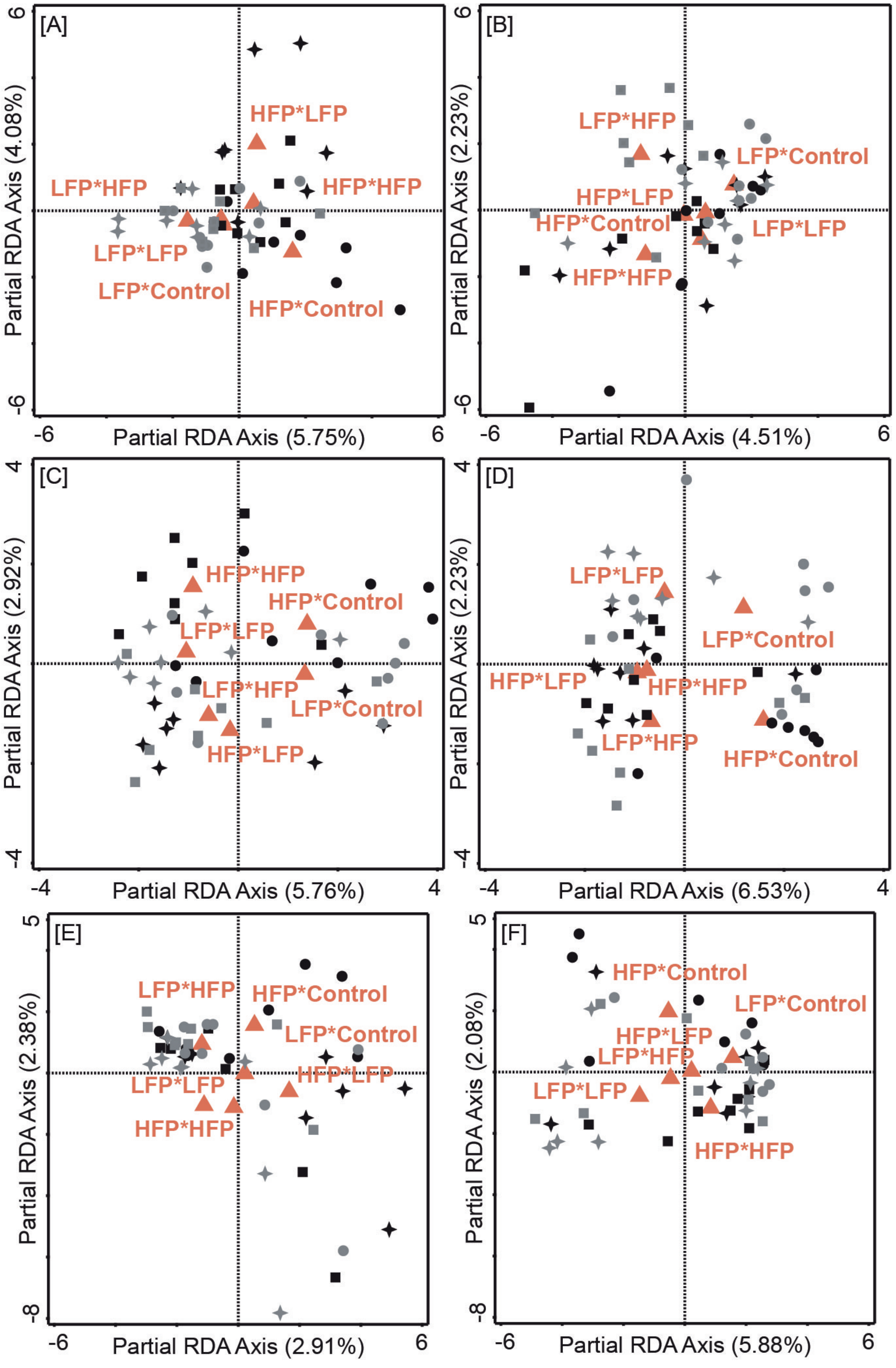

Figure 2. Biplot for partial redundancy analysis (RDA) of ileum (A and B), caecum (C and D), and colon microbiota composition (E and F) at 5 days and 2 weeks of age, respectively. Nominal environmental interaction variables are represented by red triangles. Samples are grouped by line: high feather pecking (HFP; black) and low feather pecking (LFP; grey), and by treatment: HFP (squares), Control (circles) and LFP (stars).

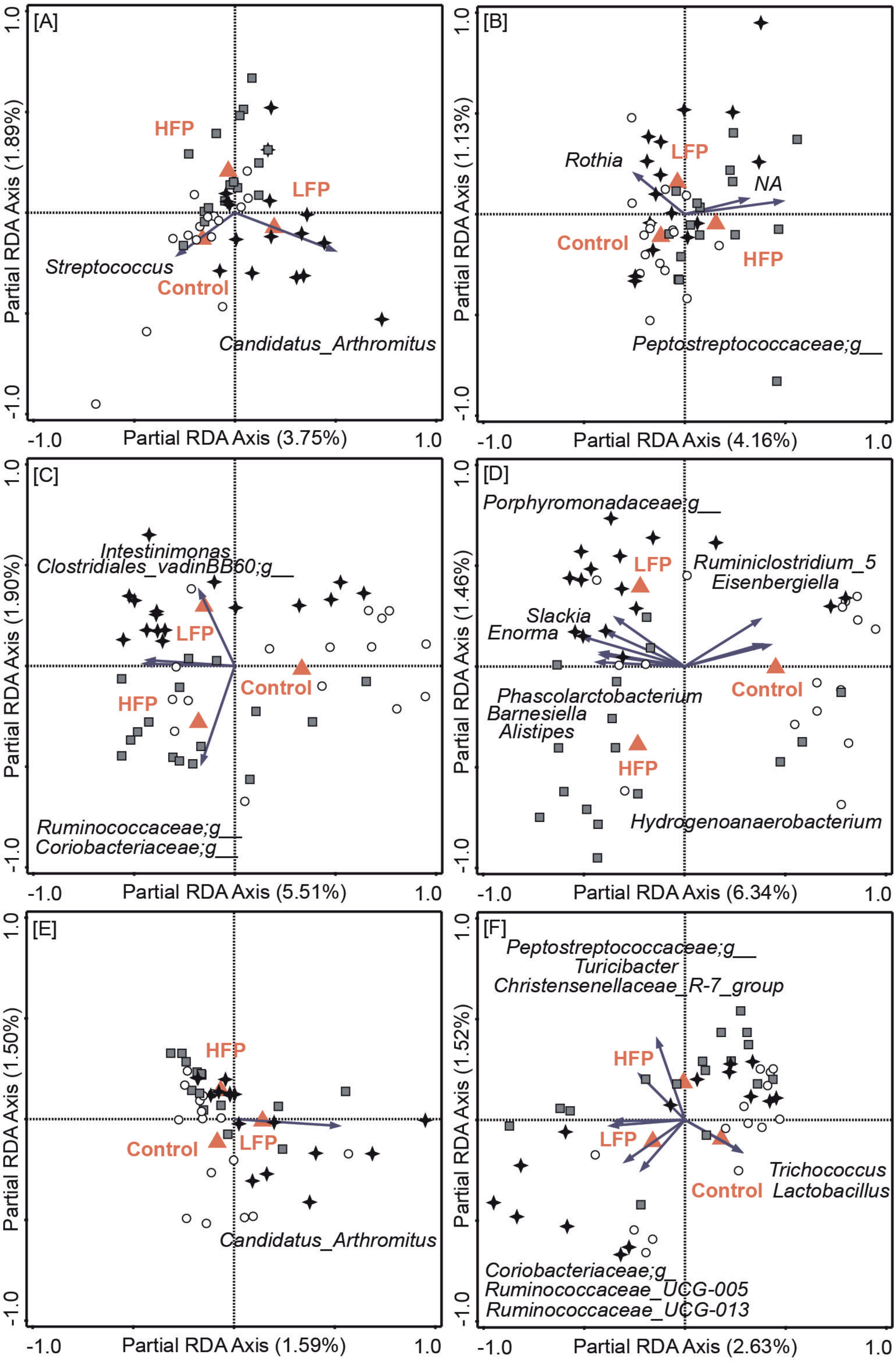

Figure 3. Triplot for partial redundancy analysis (RDA) of ileum (A and B), caecum (C and D), and colon microbiota composition (E and F) at 5 days and 2 weeks of age, respectively. Nominal environmental treatment variables are represented by red triangles. Samples are grouped by treatment: HFP (grey squares), control (white circles) and LFP (black stars). Microbial groups for which the variation in relative abundance in the data is explained for at least 15% for the caecum, and 10% for the ileum and colon by the axes are represented as vectors. Groups that could not be assigned to a specific genus are classified by the family name appended with ";g_".

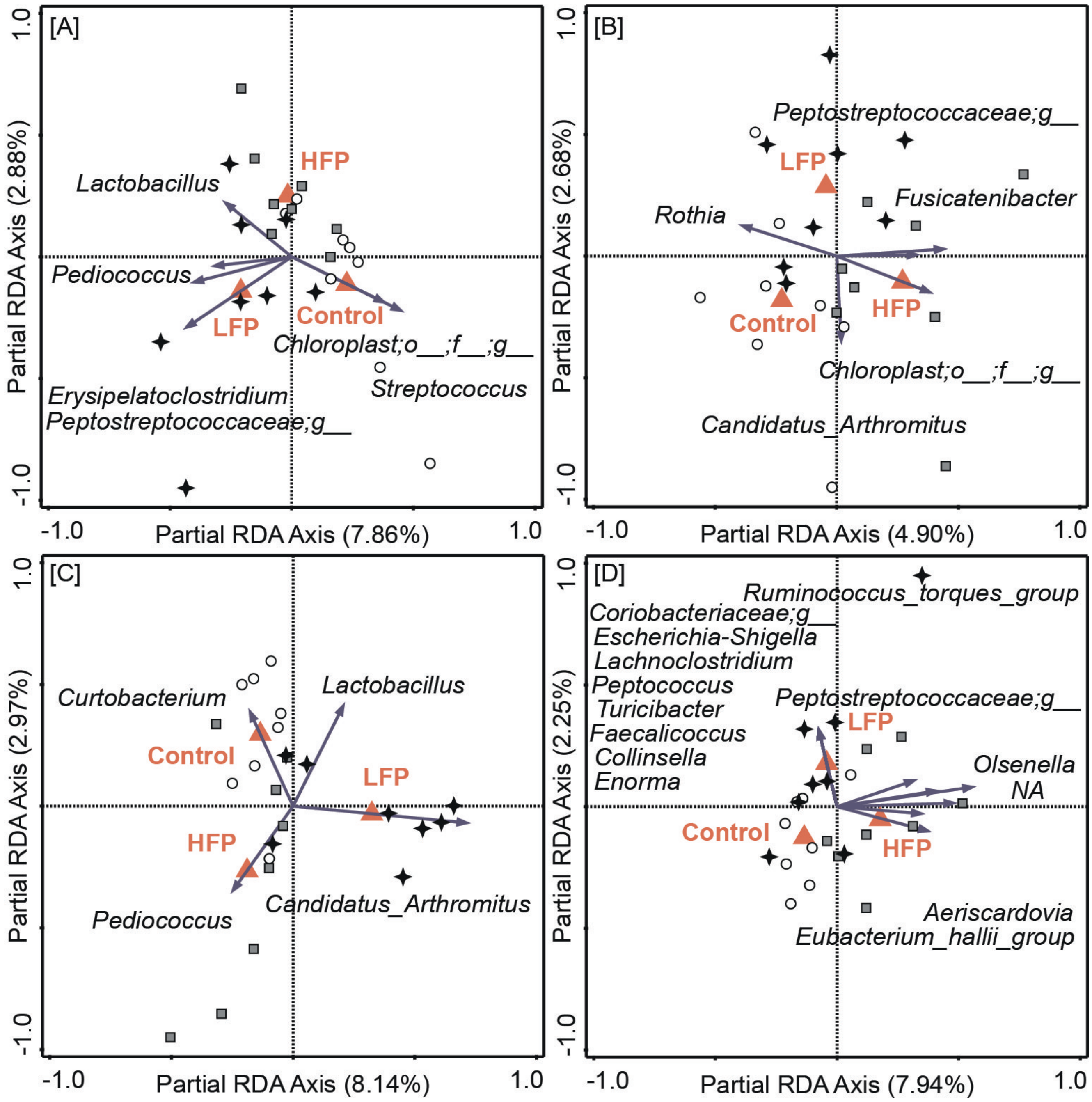

Figure 4. Triplot for partial redundancy analysis (RDA) of ileum microbiota composition of the high feather pecking (HFP; A and B) line and the low feather pecking (LFP; C and D) line at 5 days and 2 weeks of age. Nominal environmental treatment variables are represented by red triangles. Samples are grouped by treatment: HFP (grey squares), control (white circles) and LFP (black stars). Microbial groups for which the variation in relative abundance in the data is explained for at least 10% by the axes are represented as vectors. Groups that could not be assigned to a specific genus are classified by the family name appended with ";g_".

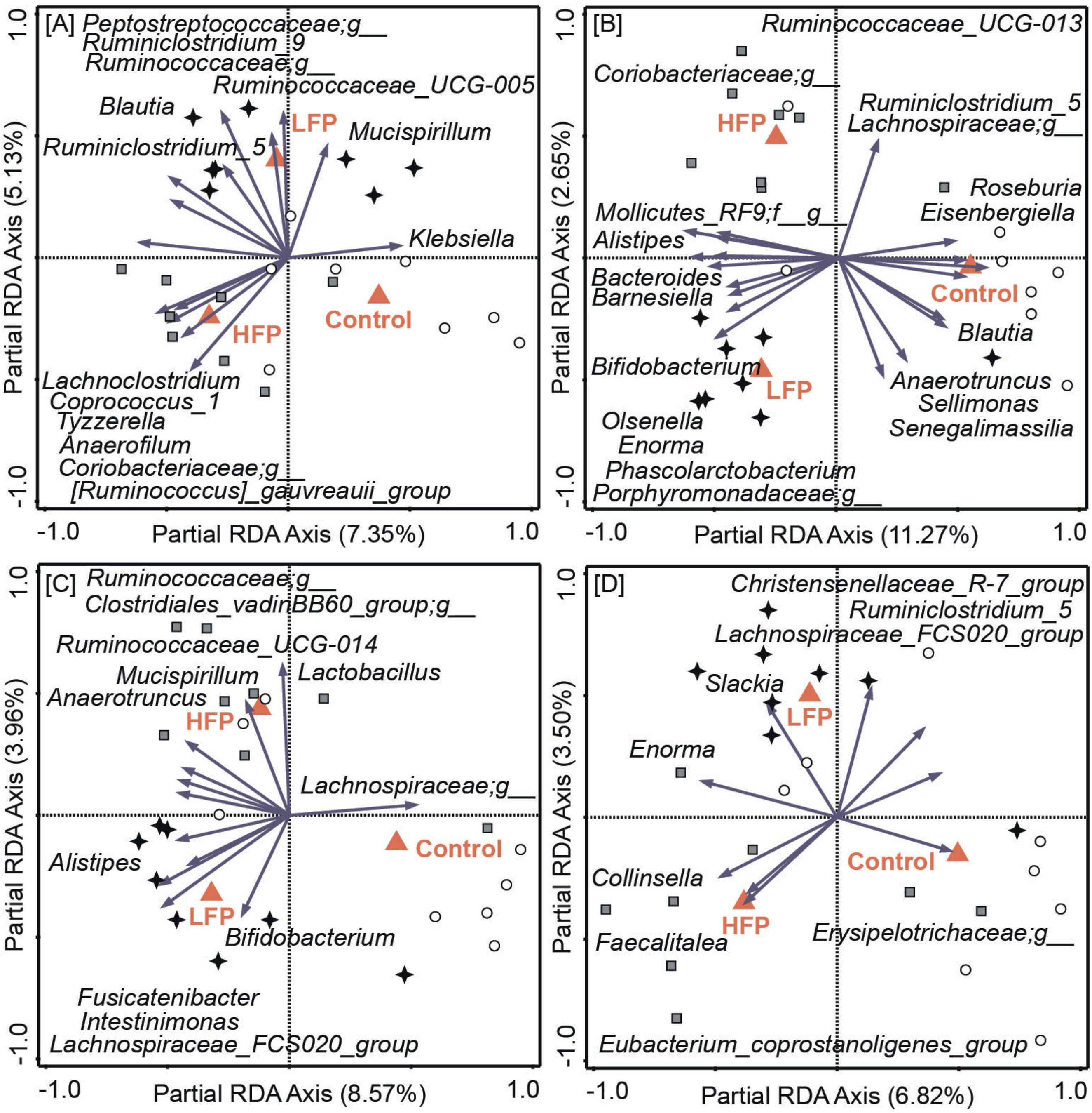

Figure 5. Triplot for partial redundancy analysis (RDA) of caecum microbiota composition of the high feather pecking (HFP; A and B) line and the low feather pecking (LFP; C and D) line at 5 days and 2 weeks of age. Nominal environmental treatment variables are represented by red triangles. Samples are grouped by treatment: HFP (grey squares), control (white circles) and LFP (black stars). Microbial groups for which the variation in relative abundance in the data is explained for at least 20% by the axes are represented as vectors. Groups that could not be assigned to a specific genus are classified by the family name appended with “;g_”.

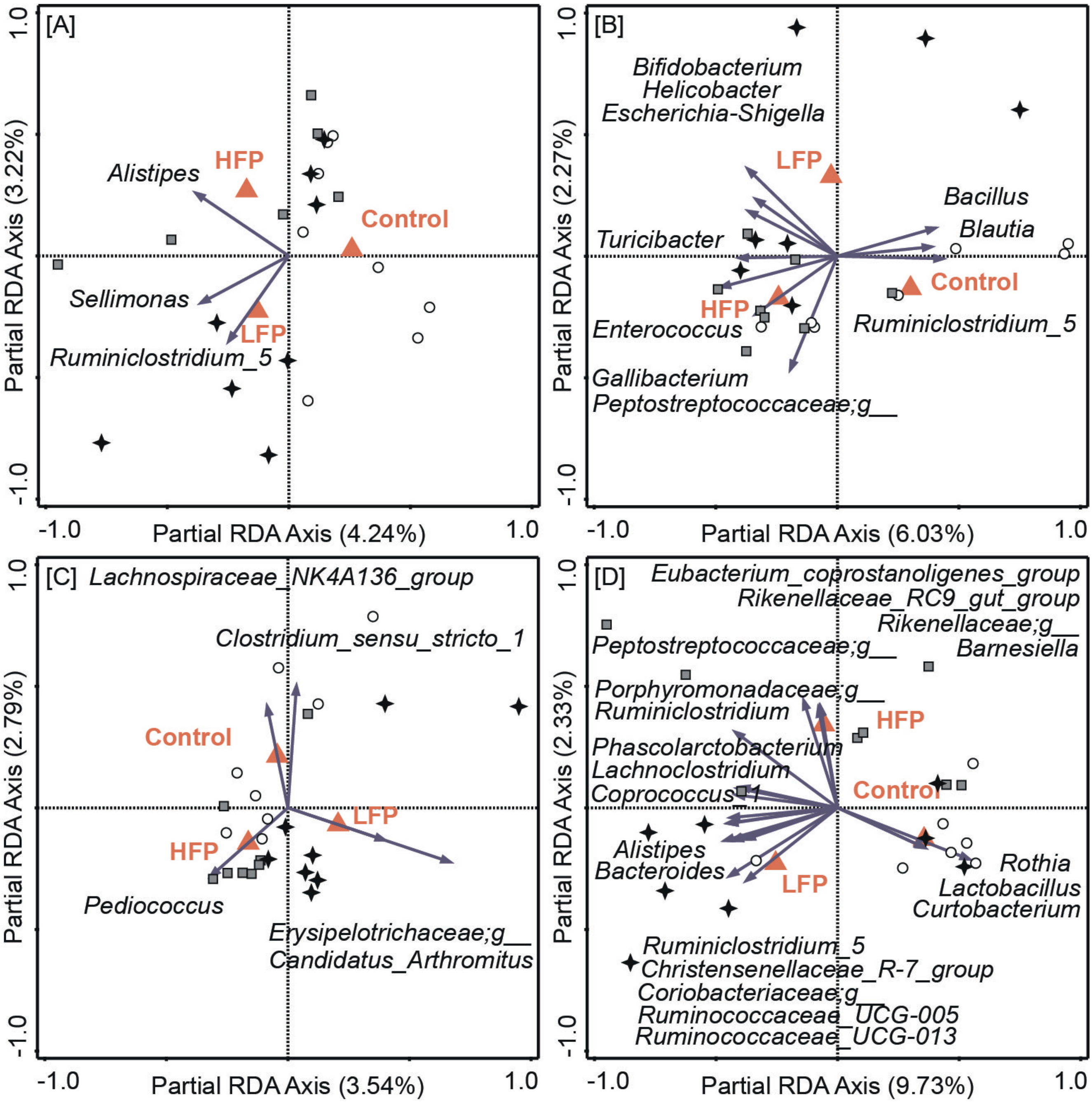

Figure 6. Triplot for partial redundancy analysis (RDA) of colon microbiota composition of the high feather pecking (HFP; A and B) line and the low feather pecking (LFP; C and D) line at 5 days and 2 weeks of age. Nominal environmental treatment variables are represented by red triangles. Samples are grouped by treatment: HFP (grey squares), control (white circles) and LFP (black stars). Microbial groups for which the variation in relative abundance in the data is explained for at least 15% by the axes are represented as vectors. Groups that could not be assigned to a specific genus are classified by the family name appended with “;g_”.

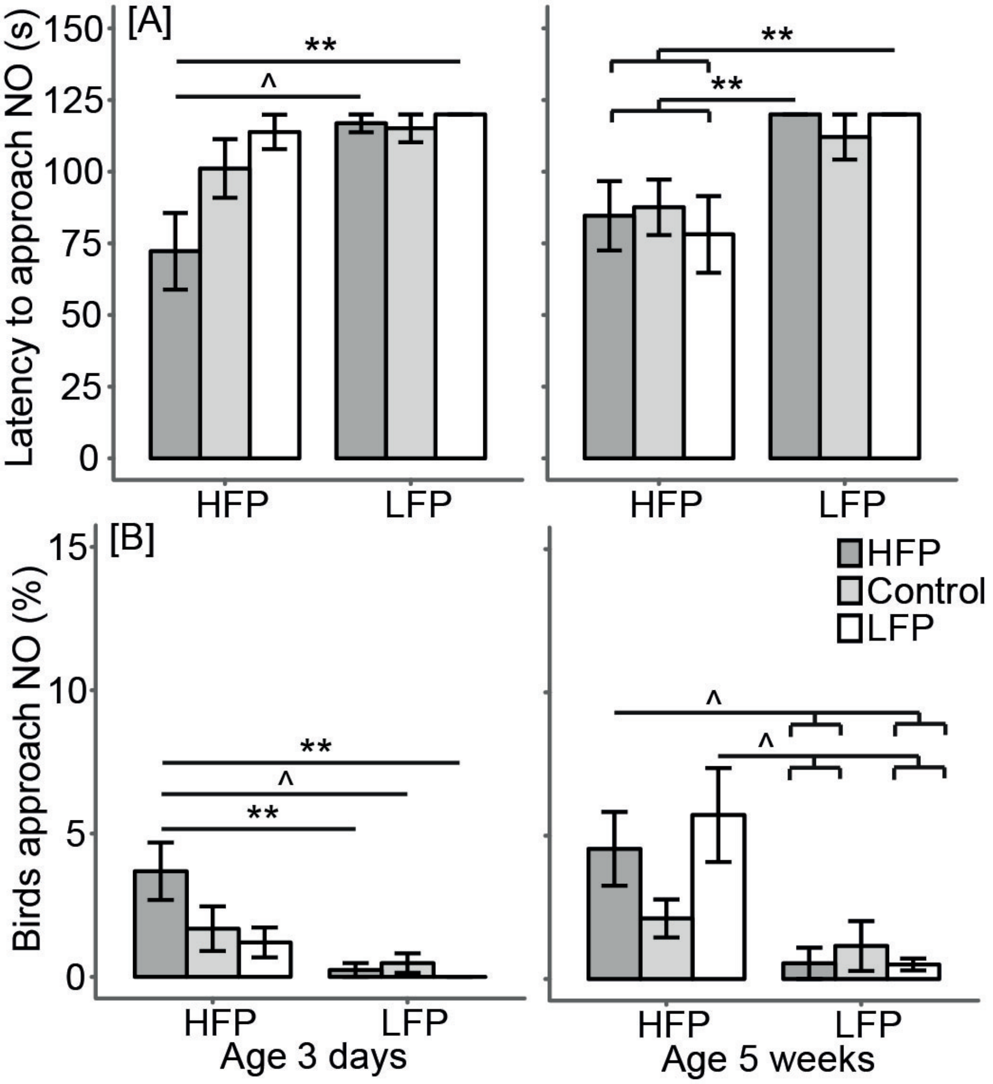

Figure 7. A) Mean latency (± SE) for three birds to approach the novel object (NO) and B) mean percentage (± SE) of birds approaching the NO at 3 days and 5 weeks of age for the high (HFP) and low feather pecking (LFP) lines treated with HFP microbiota, control treatment or LFP microbiota. ^ denotes tendencies ($P < 0.1$) and ** denotes significant differences ($P < 0.05$) between line * treatment interactions.

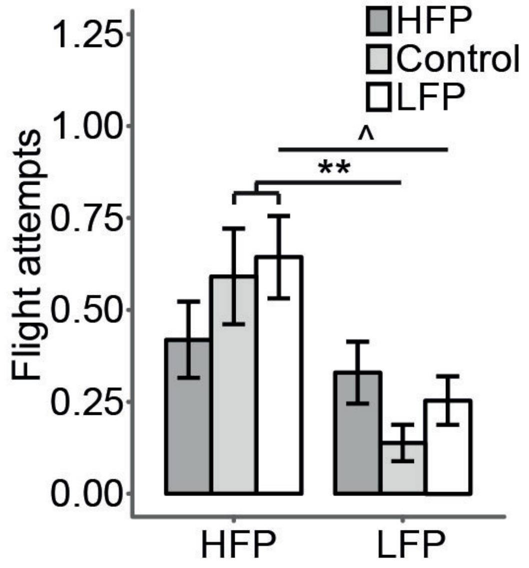

Figure 8. Mean number of flight attempts (± SE) in the novel environment test for the high (HFP) and low feather pecking (LFP) lines treated with HFP microbiota, control treatment or LFP microbiota. ^ denotes tendencies ($P < 0.1$) and ** denotes significant differences ($P < 0.05$) between line * treatment interactions.

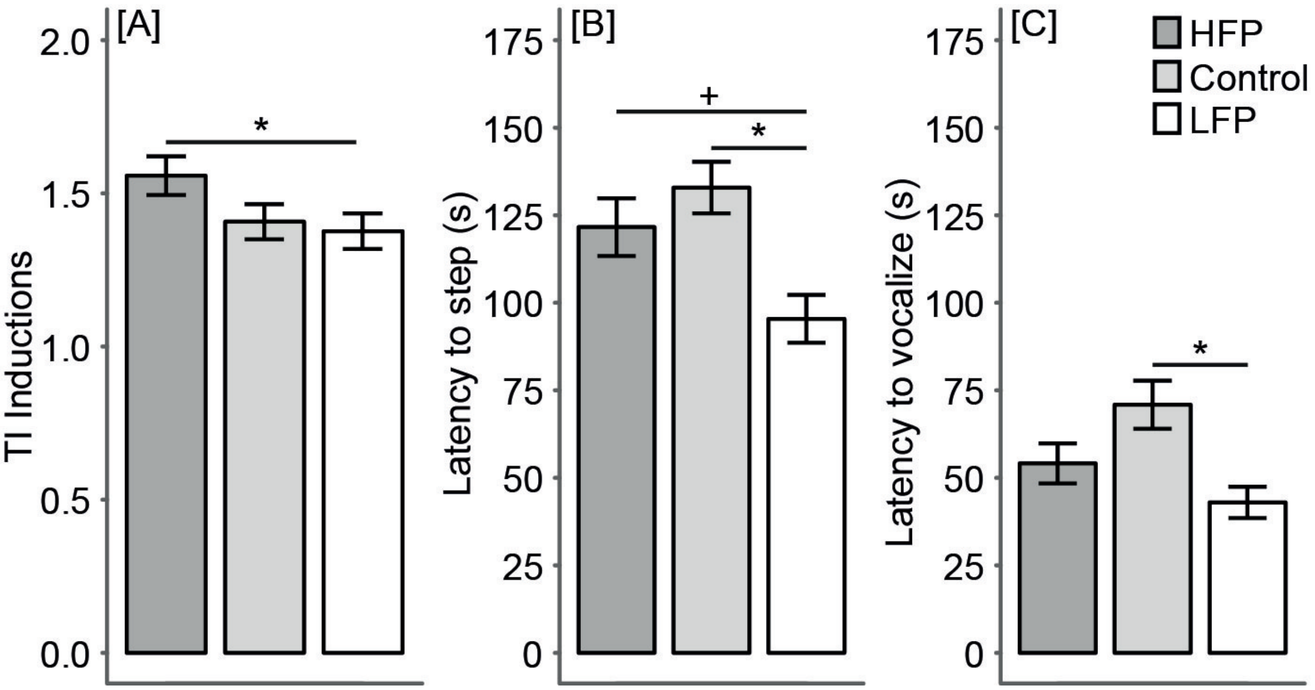

Figure 9. A) Mean number of inductions needed to reach tonic immobility (TI) (± SE) and mean latency (± SE) to B) step and C) vocalize in the open field test for birds treated with high feather pecking (HFP) microbiota, control or low feather pecking (LFP) microbiota. $^{+}$ show tendencies ($P < 0.1$) and * show significant differences ($P < 0.05$) between treatments.

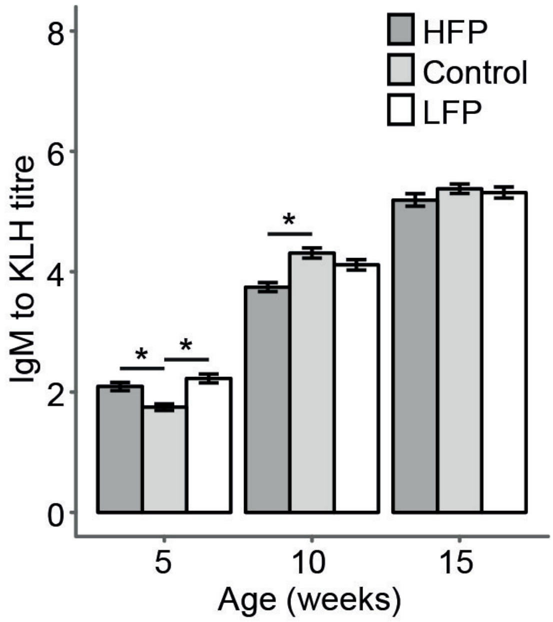

Figure 10. Mean IgM natural antibody titers to keyhole limpet hemocyanin (KLH) (± SE) at 5, 10 and 15 weeks of age for birds treated with high feather pecking (HFP) microbiota, control or low feather pecking (LFP) microbiota. * show significant differences ($P < 0.05$) between treatments.

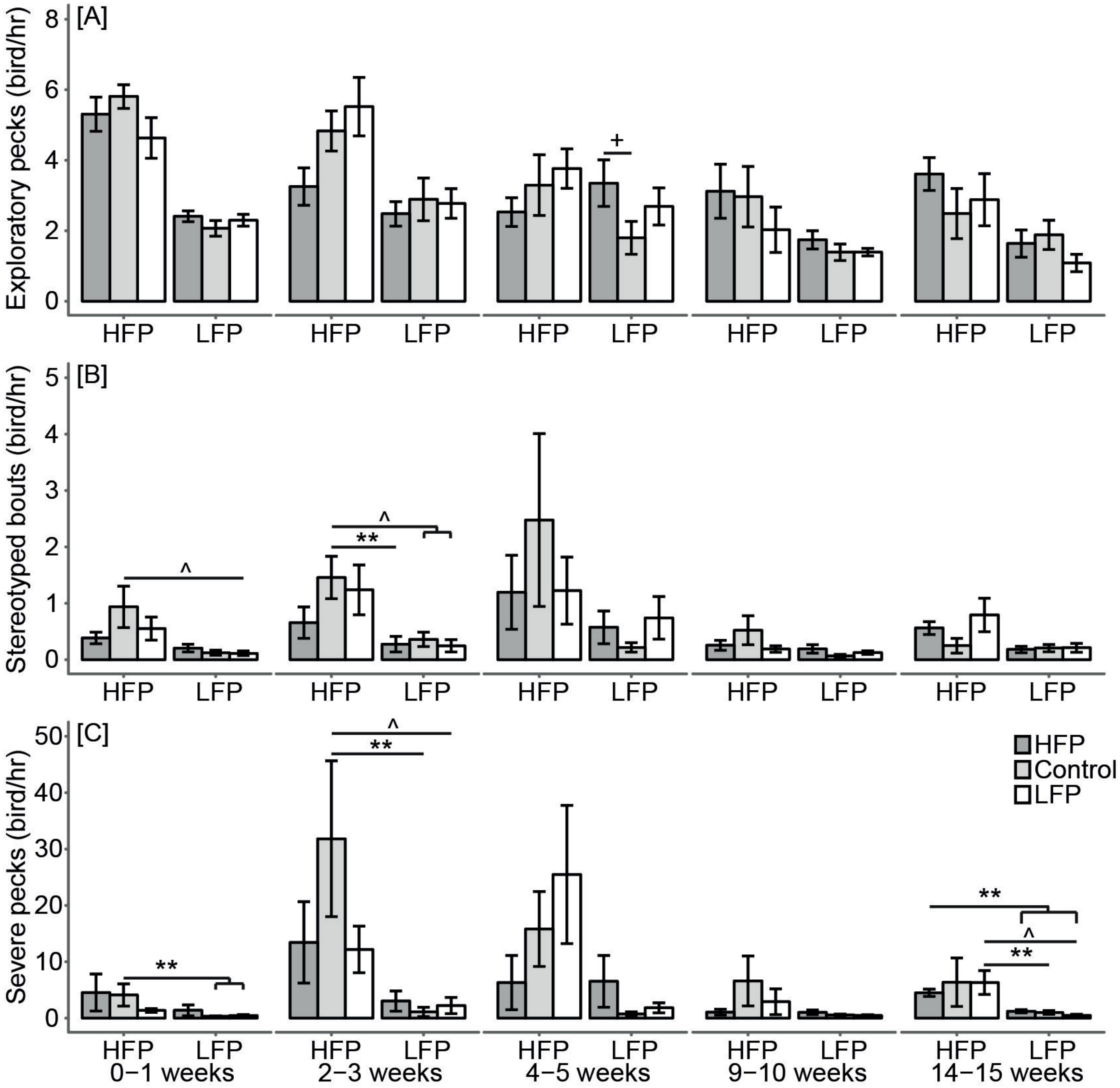

Figure 11. A) Mean exploratory pecks (± SE), B) mean stereotyped bouts (± SE) and C) mean severe pecks (± SE) per bird per hour at 0-1, 2-3, 4-5, 9-10 and 14-15 weeks of age for the high (HFP) and low feather pecking (LFP) lines treated with HFP microbiota, control or LFP microbiota. $^{+}$ show tendencies (P < 0.1) between treatments within lines; $^{\wedge}$ show tendencies (P < 0.1) and ** show significant differences (P < 0.05) between line * treatment interactions.
